# Inhibiting p38 MAPK alpha rescues axonal retrograde transport defects in a mouse model of ALS

**DOI:** 10.1038/s41419-018-0624-8

**Published:** 2018-05-22

**Authors:** Katherine L. Gibbs, Bernadett Kalmar, Elena R. Rhymes, Alexander D. Fellows, Mahmood Ahmed, Paul Whiting, Ceri H. Davies, Linda Greensmith, Giampietro Schiavo

**Affiliations:** 1 Department of Neuromuscular Disorders, UCL Institute of Neurology, University College London, London, WC1N 3BG UK; 2 GlaxoSmithKline Research and Development China, Singapore Research Centre, Singapore, 13866711 Singapore; 3 Alzheimer’s Research UK UCL Drug Discovery Institute, Gower Street, London, WC1E 6BT UK; 4 UK Dementia Research Institute at UCL, University College London, Gower Street, London, WC1E 6BT UK; 5 Discoveries Centre for Regenerative and Precision Medicine, University College London Campus, London, WC1N 3BG UK; 6 Present Address: Takeda Pharmaceutical Company Ltd, 12-10, Nihonbashi 2-chome, Chuo-ku, Tokyo Japan

## Abstract

Amyotrophic lateral sclerosis (ALS) is a fatal neurodegenerative disease caused by the degeneration of upper and lower motor neurons. Defects in axonal transport have been observed pre-symptomatically in the SOD1^G93A^ mouse model of ALS, and have been proposed to play a role in motor neuron degeneration as well as in other pathologies of the nervous system, such as Alzheimer’s disease and hereditary neuropathies. In this study, we screen a library of small-molecule kinase inhibitors towards the identification of pharmacological enhancers of the axonal retrograde transport of signalling endosomes, which might be used to normalise the rate of this process in diseased neurons. Inhibitors of p38 mitogen-activated protein kinases (p38 MAPK) were identified in this screen and were found to correct deficits in axonal retrograde transport of signalling endosomes in cultured primary SOD1^G93A^ motor neurons. In vitro knockdown experiments revealed that the alpha isoform of p38 MAPK (p38 MAPKα) was the sole isoform responsible for SOD1^G93A^-induced transport deficits. Furthermore, we found that acute treatment with p38 MAPKα inhibitors restored the physiological rate of axonal retrograde transport in vivo in early symptomatic SOD1^G93A^ mice. Our findings demonstrate the pathogenic effect of p38 MAPKα on axonal retrograde transport and identify a potential therapeutic strategy for ALS.

## Introduction

Amyotrophic lateral sclerosis (ALS) is a fatal neurodegenerative disease caused by the degeneration of both upper and lower motor neurons, resulting in progressive muscle paralysis and ultimately death. Although the precise cause of motor neuron degeneration in ALS is not yet fully understood, several mechanisms have been proposed to play a role in this process, including mitochondrial dysfunction, excitotoxicity and axonal transport deficits^1,2^. However, which of these mechanisms play a causative role in ALS pathogenesis is currently unknown^1,2^.

Deficits in axonal transport have been inferred from patient data and observed in ALS mouse models^2^. In mice overexpressing the ALS-associated human superoxide dismutase 1 G93A (SOD1^G93A^) mutant, intravital imaging in the sciatic nerve has revealed abnormalities in the axonal retrograde transport of signalling endosomes and mitochondria in pre-symptomatic mice^3^. The deficit in endosome motility was demonstrated using two independent probes: the binding fragment of tetanus toxin (H_C_T)^4^ and an antibody specific for the p75 neurotrophin receptor (αp75^NTR^)^5^. The early appearance of transport impairments in the SOD1^G93A^ mouse model^3^ suggests that these deficits play a crucial role in triggering motor neuron dysfunction, leading to the motor neuron degeneration observed in ALS.

Despite the strength of evidence demonstrating the presence of axonal transport defects in ALS^2^ and other neurodegenerative conditions^6,7^ a causal relationship between these transport impairments and neurodegeneration has not yet been shown. Indeed, the role of axonal transport defects in ALS pathogenesis remains a matter of some debate. Work using an ALS mouse model expressing the SOD1^G85R^ mutant has shown that motor neuron degeneration can also occur in the absence of overt axonal transport deficits^8^, although it should be noted that these results have been obtained using explants rather than intravital microscopy, and disease progression is much more variable in the SOD1^G85R^ mouse model than in the SOD1^G93A^ mice used in our study^3^. Hence, the identification of compounds able to specifically enhance axonal transport and thereby rescue the deficits observed in SOD1^G93A^ mice would conclusively prove the role of axonal transport defects in ALS pathogenesis.

Protein kinases have been suggested to be key players in several neurodegenerative diseases^9^. It has been proposed that disease-associated pathological proteins, such as amyloid beta (Aβ) and SOD1^G93A^, mediate their toxic effects through the activation of specific kinase cascades^10^, such as p38 mitogen-activated protein kinase (MAPK)^11–16^. In this study, we demonstrate that p38 MAPK is responsible for SOD1^G93A^-induced deficits in axonal retrograde transport in motor neurons and establish that specific inhibition of p38 MAPK alpha (p38 MAPKα) or its down-regulation corrects axonal transport deficits both in vitro and in vivo in SOD1^G93A^ mice. Inhibitors of p38 MAPKα are thus powerful tools to determine the role of axonal retrograde transport deficits in ALS pathogenesis and could be explored for future therapeutic intervention.

## Results

### Screening for pharmacological enhancers of axonal transport

The accumulation of H_C_T and α-p75^NTR^ in mouse embryonic stem (ES) cell-derived motor neurons has been previously validated in our laboratory as a biological read-out capable of identifying novel axonal trafficking effectors when combined with a siRNA screen^17,18^. In this study, we adapted this assay to screen a library of kinase inhibitors to identify novel regulators of axonal retrograde transport. As before^17,18^, transgenic HB9-GFP ES cells (HBG3) differentiated into motor neurons were used to overcome the intrinsic cellular heterogeneity of primary motor neuron cultures and obtain the large amount of neurons required for the screen. The expression of green fluorescent protein (GFP) driven by the Hb9 homeobox gene enhancer facilitated the identification of motor neurons and enabled the implementation of a reliable automatic quantification protocol^17^.

**Fig. 1:**
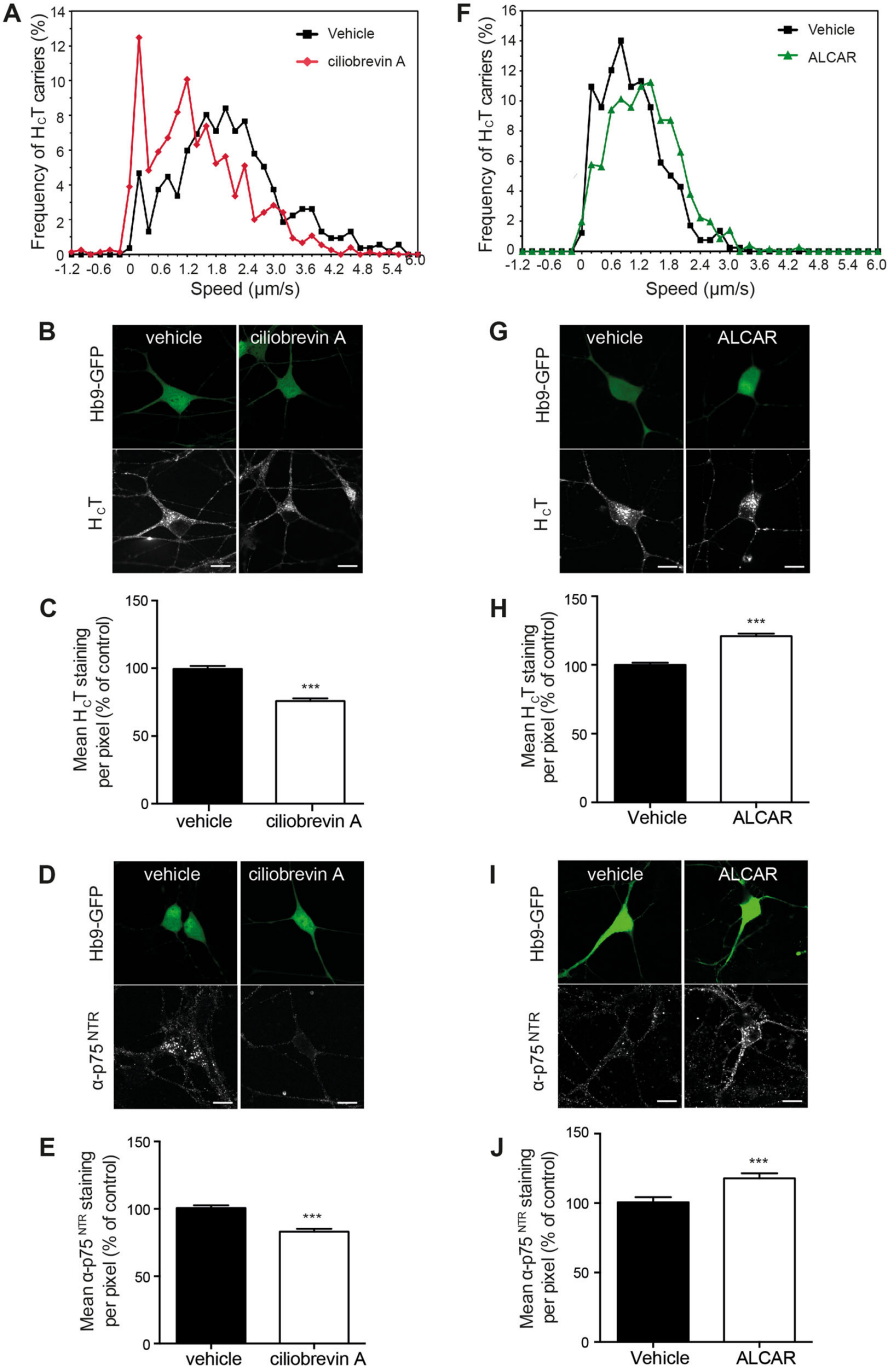
Ciliobrevin A and ALCAR modulate the axonal retrograde transport of H_C_T in motor neurons. **a** Speed profiles of H_C_T carriers in motor neurons treated with DMSO (black squares) or 50 μM ciliobrevin A (red diamonds) (DMSO: 55 carriers, 10 axons; 50 μM ciliobrevin A: 46 carriers, 8 axons; 3 independent experiments). **b, c** Effects of 100 μM ciliobrevin A on the accumulation of H_C_T in the cell body of ES cell-derived motor neurons. ES cell-derived motor neurons were incubated with AlexaFluor 555-conjugated H_C_T and DMSO or 100 μM ciliobrevin A for 2 h. Cells were then acid-washed, fixed, imaged and quantified in C as described below. **d, e** Effects of 100 μM ciliobrevin A on the accumulation of α-p75^NTR^ in the soma of ES cell-derived motor neurons. ES cell-derived motor neurons were incubated with α-p75^NTR^ and DMSO or 100 μM ciliobrevin A for 2 h. Cells were then acid-washed, fixed, permeabilised, stained for α-p75^NTR^ and quantified in E. **f** Speed profiles of H_C_T carriers in ES cell-derived motor neurons treated with control medium (black squares) or 1 mM ALCAR (green triangles) (control: 53 carriers, 10 axons; 1 mM ALCAR: 61 carriers, 8 axons; 3 independent experiments). **g, h** Effects of 1 mM ALCAR on accumulation of H_C_T (**g, h**) and α-p75^NTR^ (**i, j**) in the cell body of ES cell-derived motor neurons. ES cell-derived motor neurons were treated as described in B-E. The amount of H_C_T and α-p75^NTR^ was quantified as the mean staining intensity per pixel in the cell body. Results in C, E, H, J are expressed as a percentage of the control ± SEM (*n* = 3 independent experiments; *n* ≥ 25 cell bodies analysed per condition). *** *p* < 0.001 (unpaired Student’s t-test)

To determine whether this assay was sensitive to changes in axonal transport efficiency, we performed preliminary tests in the presence of known modulators of motor proteins involved in this process. Erythro-9-(2-hydroxy-3-nonyl)adenine (EHNA) is an established inhibitor of cytoplasmic dynein, and blocks the axonal retrograde transport of H_C_T-containing signalling endosomes^19^. Treatment of ES cell-derived motor neurons with 1 mM EHNA resulted in a significant decrease in H_C_T or α-p75^NTR^ accumulation in the soma (Supplementary Fig. 1A-D), indicating that the sensitivity of this assay is sufficient to detect alterations in axonal retrograde transport rates. However, EHNA is not a very selective inhibitor^20,21^ and therefore we cannot rule out the possibility that its effects on the somatic accumulation of H_C_T and α-p75^NTR^ may be due to off-target effects. We therefore incubated wild-type primary motor neurons with 50 μM ciliobrevin A, a blocker of the ATPase activity of cytoplasmic dynein^22^. In vitro axonal transport assays revealed a significant inhibition of the retrograde transport of H_C_T-labelled organelles (Fig. 1a), whilst 100 µM ciliobrevin A caused a complete block of transport. Importantly, this inhibition of axonal transport was also mirrored by a significant reduction of H_C_T (Fig. 1b, c**)** and α-p75^NTR^ (Fig. 1d, e) accumulation in the soma.

Few specific enhancers of axonal transport are currently available. Acetyl-L-carnitine (ALCAR) was previously reported to enhance axonal retrograde transport in diabetic rats^23^. Accordingly, treatment of ES cell-derived motor neurons with 1 mM ALCAR increased the retrograde transport speed of H_C_T-labelled organelles in cultured motor neurons (Fig. 1f). We were also consistently able to detect this increase in transport efficiency using the accumulation assay, with approximately 25–30% more accumulation of H_C_T (Fig. 1g,h) and α-p75^NTR^ (Fig. 1i,j) in ALCAR-treated motor neurons.

A further validation step for this assay was to confirm whether this approach could detect the axonal transport deficits previously observed in SOD1^G93A^ motor neurons^24^. Using primary motor neurons cultures, we found a significant decrease in H_C_T accumulation in SOD1^G93A^ motor neurons compared to wild-type controls (Supplementary Fig. 1E, F).

**Fig. 2:**
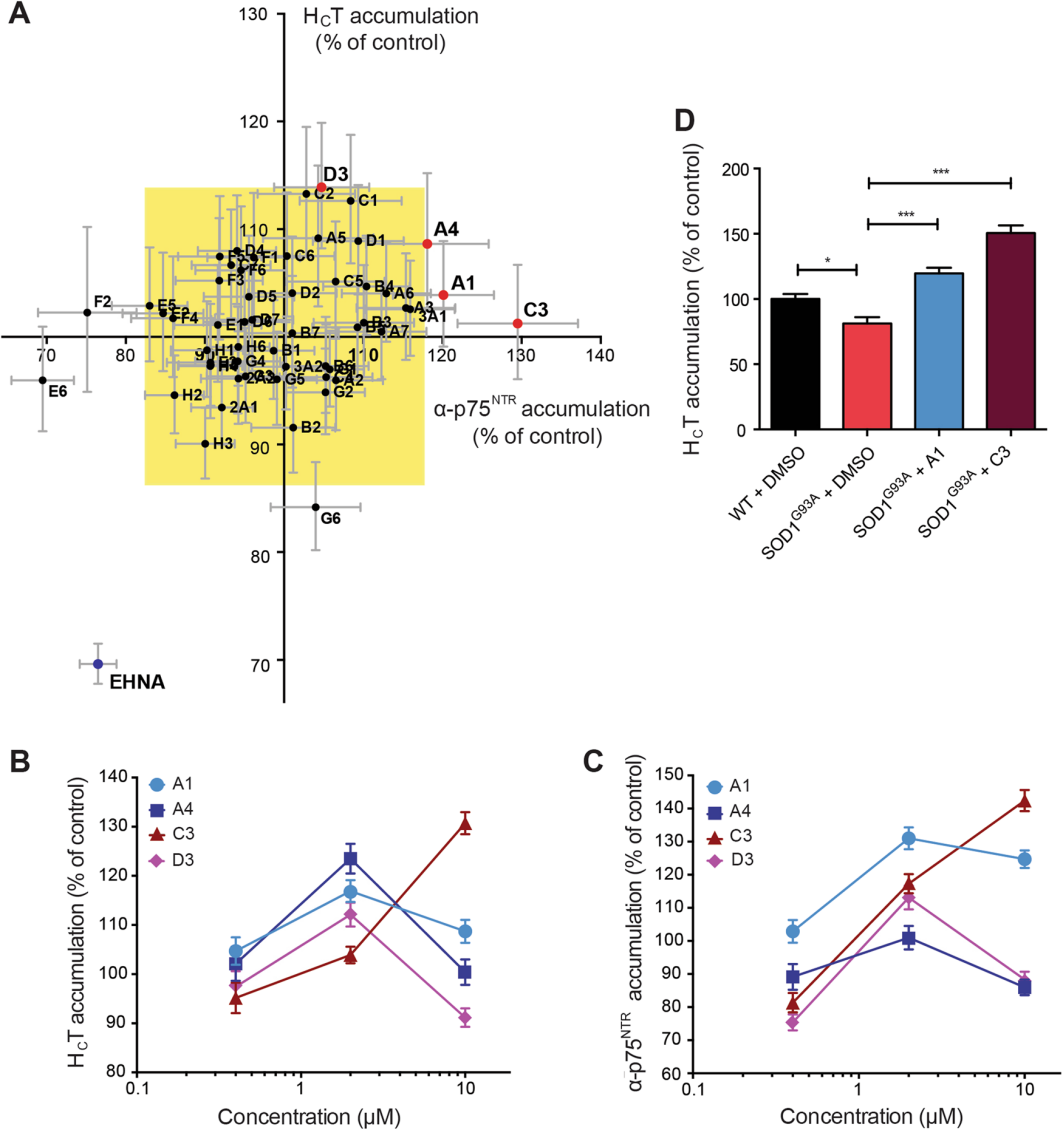
Chemical screen for the identification of enhancers of H_C_T and α-p75^NTR^ accumulation in the soma of ES cell-derived motor neurons. **a** The results of a small-molecule chemical screen are shown as an XY plot of the normalized mean staining intensity for α-p75^NTR^ versus AlexaFluor 555-conjugated H_C_T. α-p75^NTR^ was detected using an AlexaFluor 647-conjugated donkey anti-rabbit secondary antibody. Active compounds (red dots) were defined as those able to increase accumulation of α-p75^NTR^ and H_C_T by at least three times the standard deviation of the dataset (represented by the yellow rectangle). The negative control (EHNA) is shown in blue (*n* ≥ 25 cell bodies analysed per condition; *n* = 3 repeats). **b, c** Dose–response analysis of the top four active compounds identified by the screening displayed in A. The accumulation assay was performed at 0.4, 2 and 10 μM and the amount of H_C_T (**b**) and α-p75^NTR^ (**c**) in the soma was quantified as the mean staining intensity per pixel ( ± SEM) (*n* ≥ 25 cell bodies per condition, *n* = 3 repeats). **d** Effects of 2 μM A1 and C3 on the accumulation of H_C_T in the cell body of primary motor neurons isolated from E13 SOD1^G93A^ embryos (DIV6). The amount of H_C_T was quantified as described in B, C and shown as mean ± SEM. Significance levels were determined using the Kruskal–Wallis test followed by Dunn’s multiple comparisons test. *n* = 3 repeats; *** *p* < 0.001; * *p* < 0.05

Having established that the accumulation assay was suitable for the detection of changes in axonal retrograde transport efficiency induced both pharmacologically and by the expression of pathogenic proteins, we used it to screen a library of small-molecule kinase inhibitors (Supplementary Table 1). Compounds were initially tested at a concentration of 2 μM and their effects compared with vehicle (DMSO). Active compounds were defined as those that increased the mean staining intensity of H_C_T and α-p75^NTR^ by at least three standard deviations above the control level (yellow rectangle; Fig. 2a and Supplementary Fig. 2A), which was taken as 100%. Two active compounds (A1 and C3) were identified (Fig. 2a**)**, representing a primary hit rate of 4%. We also identified three inhibitory molecules (E6, F2 and G6; Fig. 2a), which however were not investigated further.

We next performed dose–response assays using the same methodology. In addition to the previously tested concentration of 2 μM, the assay was also performed at 0.4 and 10 μM (Fig. 2b, c). Two additional compounds from the initial screen (A4, D3) were included in the dose–response assays because their effects on H_C_T and α-p75^NTR^ accumulation were close to the significance boundary (yellow rectangle; Fig. 2a) and their interesting pharmacological target (Supplementary Table 1), raising the possibility that the concentration used in the first screen might have been too low. All four compounds were found to modulate the accumulation of H_C_T and α-p75^NTR^ at doses higher than 0.4 μM (Fig. 2b, c). However, at 10 μM, these molecules (with the exception of C3) had either a toxic and/or an inhibitory effect on H_C_T and α-p75^NTR^ accumulation (Fig. 2b, c).

Based on the dose–response data, compounds A1 and C3 were selected for further analyses. Both molecules were able to correct the deficits in H_C_T accumulation observed in SOD1^G93A^ motor neurons when tested at 2 μM (Fig. 2d). However, upon further testing, compound C3 was also found to cause axonal blebbing (data not shown) and was therefore excluded from further studies.

### Inhibition of p38 MAPK corrects axonal transport defects in vitro

**Fig. 3:**
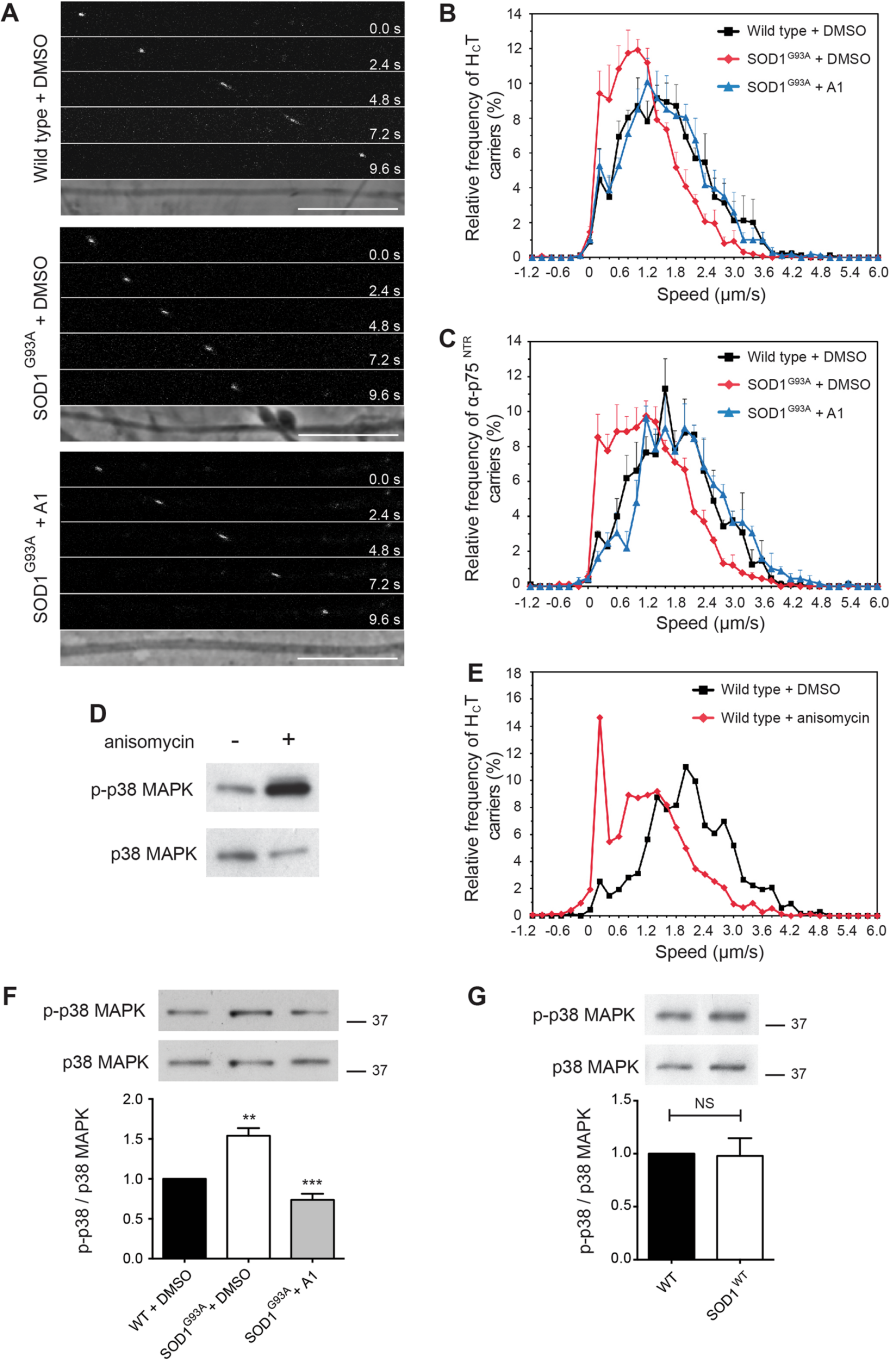
Impaired axonal retrograde transport can by normalised by pre-treatment with a p38 MAPK inhibitor in embryonic SOD1^G93A^ motor neurons. **a** Frames from confocal time series of H_C_T-labelled endosomes in wild type + DMSO (top), SOD1^G93A^ + DMSO (middle) and SOD1^G93A^ + 2 μM A1 (bottom) axons. Scale bars, 10 μm. **b** Speed profiles of H_C_T carriers in wild type + DMSO (black squares), SOD1^G93A^ + DMSO (red diamonds) and SOD1^G93A^ + 2 μM A1 (blue triangles) motor neuron cultures. Comparison of the curves reveals SOD1^G93A^ motor neurons treated with compound A1 have a similar speed distribution to wild-type cultures (wild type: 94 carriers, 13 axons; SOD1^G93A^: 126 carriers, 14 axons; SOD1^G93A^ + 2 μM A1: 121 carriers, 16 axons; 4 independent experiments; data shown as mean ± SEM). **c** Speed distribution profiles of α-p75^NTR^ carriers in wild type + DMSO, SOD1^G93A^ + DMSO (vehicle control) and SOD1^G93A^ + 2 μM A1 motor neuron cultures (wild type: 118 carriers, 6 axons; SOD1^G93A^: 85 carriers, 6 axons; SOD1^G93A^ + 2 μM A1: 96 carriers, 8 axons; 3 independent experiments; data shown as mean ± SEM). **d** Treatment of wild-type motor neuron cultures with 0.5 μg/ml anisomycin (1.9 μM) for 30 min causes a substantial activation of p38 MAPK (upper panel; detected with a phosphospecific pT180/pY182 α-p38 MAPK antibody). The total content of p38 MAPK in the samples is shown in the lower panel (pan α-p38 MAPK antibody). **e** Speed profiles of H_C_T carriers in motor neuron cultures treated with either DMSO (black squares) or 1.9 μM anisomycin (red diamonds). Anisomycin causes a shift to slower transport speeds (DMSO treated: 77 carriers, 8 axons; anisomycin treated: 76 carriers, 8 axons; 3 independent experiments; data shown as mean ± SEM). **f** Western blot showing activation of p38 MAPK in primary SOD1^G93A^ motor neuron cultures compared to wild-type controls. Compound A1 normalises phospho-p38 MAPK in SOD1^G93A^ cultures to wild-type levels (*top)*. The upper panel was stained as in panel D. Quantification reveals a 1.5 fold increase in phospho-p38 MAPK in SOD1^G93A^ motor neuron cultures compared to wild-type cells, and confirms the normalisation of p38 MAPK activity by compound A1 *(bottom)* (*n* = 3 independent experiments). Data shown as mean ± SEM. ** *p* < 0.01, *** *p* < 0.001 (one-way ANOVA followed by Sidak’s multiple comparison test). **g** Western blot showing active p38 MAPK in motor neurons overexpressing the SOD1^WT^ protein compared to wild-type controls *(top)*. Western blot quantification detected no significant difference in p38 MAPK activation between wild-type cultures and motor neurons overexpressing SOD1^WT^ *(bottom)* (*n* = 3 independent experiments). Data shown as mean ± SEM. Non-significant (NS) using unpaired Student’s t-test

Compound A1 (SB-239272; CHEMBL275798) is an inhibitor of p38 MAPK. Interestingly, abnormal activation of p38 MAPK has been previously implicated in ALS pathogenesis^11–16^ and was found to inhibit axonal transport in squid axoplasm^12,25^. We found that compound A1 at 2 μM was able to correct deficits in the axonal retrograde transport of both H_C_T and α-p75^NTR^ in SOD1^G93A^ motor neurons (Fig. 3a–c). In contrast, when applied to wild-type motor neurons, compound A1 had no effect (Supplementary Fig. 2B).

To confirm that activated p38 MAPK is a negative regulator of axonal transport, we next performed live in vitro axonal transport assays in the presence of 1.9 μM anisomycin, an activator of p38 MAPK^26^ (Fig. 3d). Anisomycin-treated wild-type motor neurons displayed a strong inhibition of the retrograde transport of H_C_T (Fig. 3e), similar to that observed in untreated SOD1^G93A^ motor neurons (Fig. 3b).

We then tested additional kinase inhibitors in the accumulation assay to further validate p38 MAPK as the target of compound A1 (Supplementary Table 2**)**. Four chemically diverse p38 MAPK inhibitors were found to increase H_C_T and α-p75^NTR^ accumulation in ES cell-derived motor neurons (Supplementary Fig. 3A and Supplementary Table 3), and correct SOD1^G93A^-induced deficits in axonal transport, mimicking the effects of compound A1 (Supplementary Fig. 3B-E). These results strongly suggest that these compounds normalise axonal retrograde transport in SOD1^G93A^ motor neurons via the inhibition of p38 MAPK.

### p38 MAPK is activated in motor neurons isolated from SOD1^G93A^ mice

We next investigated whether activated p38 MAPK can be detected in embryonic primary SOD1^G93A^ motor neurons. As shown in Fig. 3f, a significant activation of p38 MAPK was found in SOD1^G93A^ motor neuron cultures compared to wild-type controls and motor neurons overexpressing wild-type human SOD1 (SOD1^WT^) (Fig. 3g). We also assessed the levels of activated p38 MAPK in the spinal cords of mice overexpressing either SOD1^G93A^ or SOD1^WT^ at different disease stages (Supplementary Fig. 4). Pre-symptomatic (36 d), early symptomatic (73 d), symptomatic (96 d) and late symptomatic (115 d) mice were chosen so that the level of activated p38 MAPK could be directly correlated with the in vivo axonal transport defects previously described^3^. p38 MAPK was found to be activated in the spinal cord of pre-symptomatic SOD1^G93A^ mice compared to wild type and SOD1^WT^ controls (Supplementary Fig. 4). Crucially, p38 MAPK activity was highest in early symptomatic SOD1^G93A^ mice—the age at which in vivo axonal transport defects are most severe^3^. These data are in agreement with previous findings demonstrating activation of p38 MAPK in SOD1^G93A^ mice^12,13,15^.

### p38 MAPKα activation causes SOD1^G93A^-induced deficits in axonal transport

**Fig. 4:**
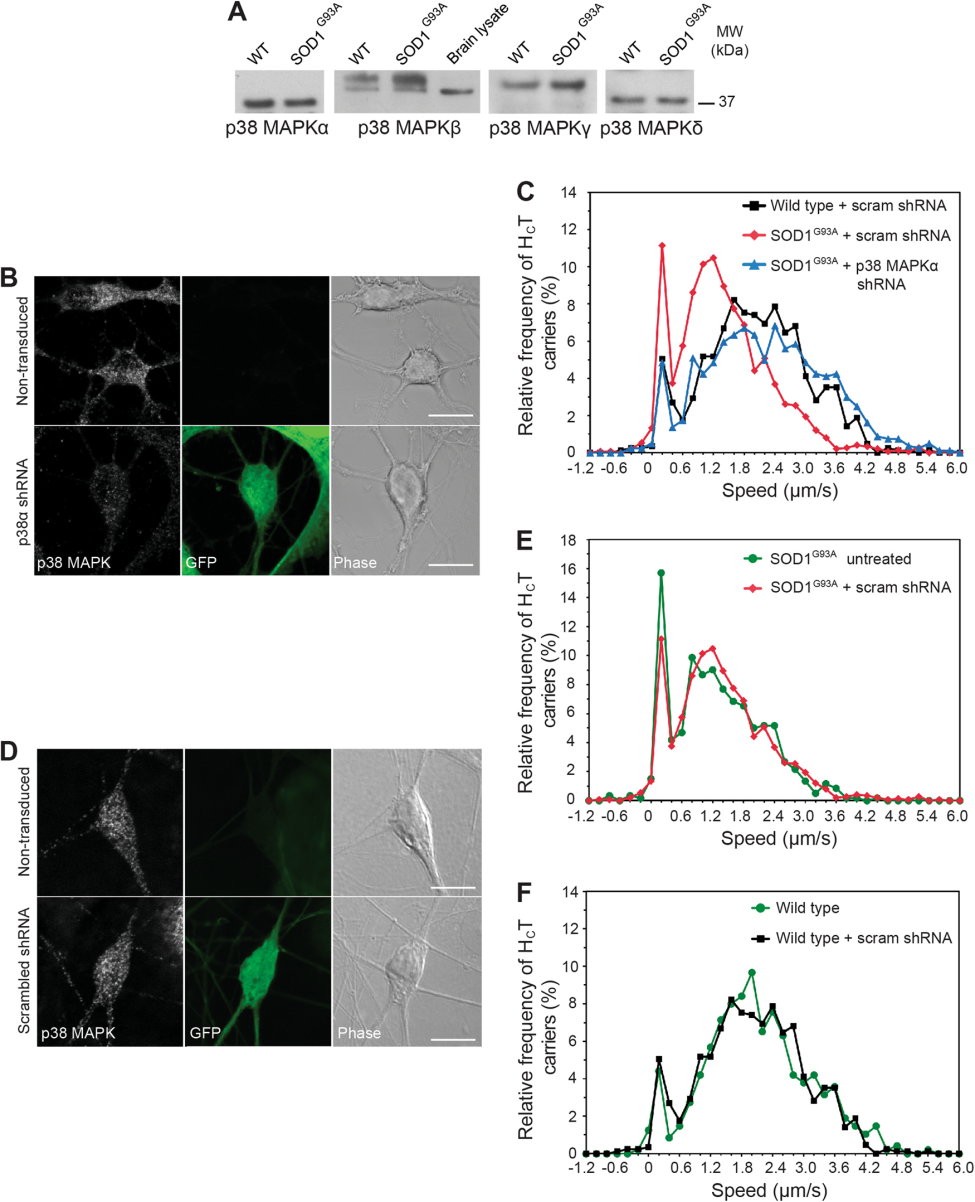
p38 MAPKα knockdown normalises axonal retrograde transport in SOD1^G93A^ motor neurons. **a** Western blot analysis reveals all four p38 MAPK isoforms (α, β, γ and δ) are expressed in wild type and SOD1^G93A^ motor neuron lysates. Mouse brain lysate was loaded as an antibody control for p38 MAPKβ. The upper band is only detected in spinal cord extracts and may represent a posttranslational modified form of p38 MAPKβ. **b** Lentiviral delivery of shRNA targeted against p38 MAPKα reduces its levels in primary motor neurons. Transduced motor neurons express GFP. **c** Speed profiles of H_C_T-containing signalling endosomes in wild-type motor neurons transduced with scrambled shRNA (black squares), SOD1^G93A^ motor neurons transduced with scrambled shRNA (red diamonds) and SOD1^G93A^ motor neurons transduced with p38 MAPKα shRNA (blue triangles). Axonal transport has been quantified only in GFP-positive motor neurons. Comparison of the curves reveals that knocking down p38 MAPKα in SOD1^G93A^ motor neurons (blue triangles) accelerates axonal transport to wild-type speeds (black squares) (wild type + scrambled shRNA: 89 carriers, 17 axons; SOD1^G93A^ + scrambled shRNA: 104 carriers, 24 axons; SOD1^G93A^ + p38 MAPKα shRNA: 99 carriers, 20 axons; 4 independent experiments). **d** Lentiviral delivery of scrambled shRNA has no effect on levels of p38 MAPKα in primary motor neurons. Transduced motor neurons express GFP. **e** Speed profiles of H_C_T carriers in untreated SOD1^G93A^ motor neurons (green circles) and SOD1^G93A^ motor neurons transduced with scrambled shRNA (scram; red diamonds). Comparison of the curves reveals that the scrambled shRNA construct (red diamonds) has no effect on axonal transport speeds (SOD1^G93A^ untreated: 45 carriers, 7 axons; SOD1^G93A^ + scrambled shRNA: 104 carriers, 24 axons; 4 independent experiments). **f** Speed profiles of H_C_T carriers in untreated wild-type motor neurons (green circles) and wild-type motor neurons transduced with scrambled shRNA (scram; black squares). Comparison of the curves reveals that scrambled shRNA constructs have no effect on axonal transport speeds (wild-type untreated: 62 carriers, 5 axons; wild type + scrambled shRNA: 89 carriers, 17 axons; 3 independent experiments)

The four isoforms of p38 MAPK (α, β, γ, δ) are all expressed in primary motor neurons (Fig. 4a). Because p38 MAPKs are involved in numerous signalling pathways, it is crucial to determine which isoform(s) is responsible for the transport inhibition observed in SOD1^G93A^ motor neurons. This in turn would provide a more specific therapeutic target reducing undesirable side effects, a problem which has severely hampered the development of new therapeutic approaches directed towards p38 MAPK^27^.

Since p38 MAPKα has been previously shown to regulate anterograde transport in isolated squid axoplasm^12^, we sought to test whether this isoform is responsible for the defects in axonal retrograde transport observed in SOD1^G93A^ mice. Lentiviral shRNA vectors expressing GFP as a reporter were used to knockdown p38 MAPKα in SOD1^G93A^ motor neurons (Fig. 4b and Supplementary Fig. 5A) and restored axonal retrograde transport to physiological wild-type levels (Fig. 4c), mimicking the results obtained by pharmacological inhibition of p38 MAPK. Transduction of motor neurons with a scrambled shRNA construct had no effect on p38 MAPKα expression (Fig. 4d and Supplementary Fig. 5B), or on axonal transport speeds, in either SOD1^G93A^ (Fig. 4c, e) or wild-type motor neurons (Fig. 4c, f). To confirm that these results were specific to p38 MAPKα, we also knocked down p38 MAPKδ (Supplementary Fig. S5C) in SOD1^G93A^ motor neurons, and observed no effect on axonal retrograde transport speeds (Supplementary Fig. S5D).

### SB-239063 corrects axonal transport defects in vitro and in vivo

**Fig. 5:**
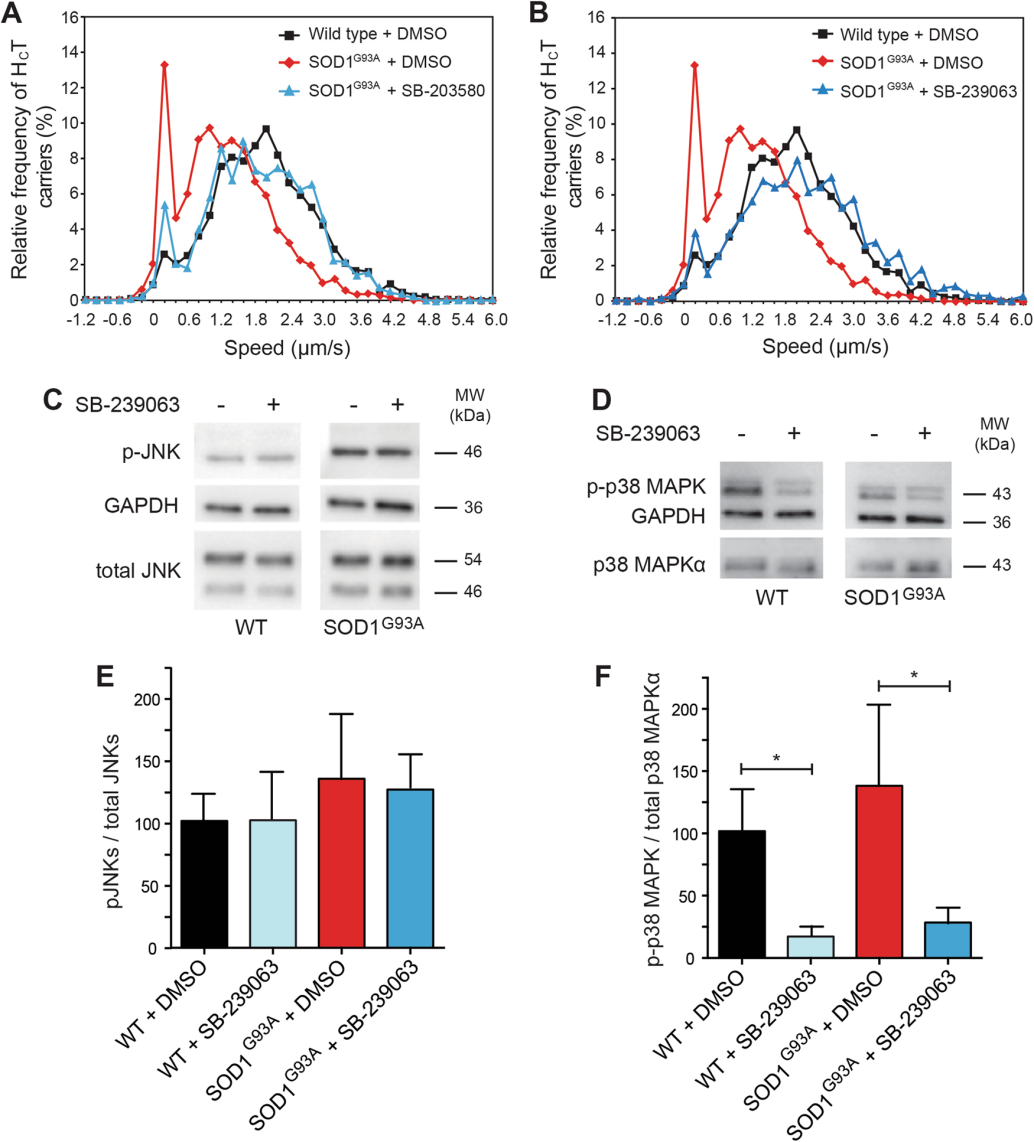
Rescue of axonal transport deficits in SOD1^G93A^ motor neurons is independent of JNK inhibition. **a** Speed profiles of H_C_T carriers in wild-type motor neurons treated with DMSO (black squares), SOD1^G93A^ motor neurons treated with DMSO (red diamonds) and SOD1^G93A^ motor neurons treated with 2 μM SB-203580 (blue triangles). Comparison of the curves reveals SOD1^G93A^ motor neurons treated with 2 μM SB-203580 have a similar speed distribution to wild-type cultures (wild type: 134 carriers, 16 axons; SOD1^G93A^: 112 carriers, 12 axons; SOD1^G93A^ + 2 μM SB-203580: 75 carriers, 10 axons; 3 independent experiments). **b** Speed distribution profiles of H_C_T carriers in wild-type motor neurons treated with DMSO (black squares), SOD1^G93A^ motor neurons treated with DMSO (red diamonds) and SOD1^G93A^ motor neurons treated with 2 μM SB-239063, a p38 MAPKα and β specific inhibitor (blue triangles). Comparison of the speed profiles reveals SOD1^G93A^ motor neurons treated with 2 μM SB-239063 have a similar speed distribution curve to wild-type cultures (wild type: 134 carriers, 16 axons; SOD1^G93A^: 112 carriers, 12 axons; SOD1^G93A^ + 2 μM SB-239063: 85 carriers, 9 axons; 3 independent experiments). **c**–**f** The rescue of deficits in the axonal transport of signal endosomes by SB-239063 is independent of JNK inhibition. Wild type (WT) and SOD1^G93A^ motor neuron lysates were blotted and stained for active phosphorylated JNK (p-JNK) and for total JNK (**c**). The ratio of these signals shown in (**e**) is not significantly affected by treatment with 2 μM SB-239063. These samples were stained for phosphorylated p38 MAPK (p-p38 MAPK), total p38 MAPKα and GAPDH as loading control (**d**). The ratio of p-p38 MAPK / total p38 MAPKα signals shown in (**f**) reveals a significant inhibition of p38 MAPK upon SB-239063 treatment even in conditions maximising p38 MAPK activation (0.5 μ./ml anisomycin for 60 min; see Fig. 3). *n* = 3 independent experiments; data shown as mean ± SEM. *, *p* < 0.05 (two-way ANOVA)

We next looked to confirm the role of p38 MAPKα on axonal transport in vitro and in vivo using established pharmacological tools. SB-203580 is an inhibitor of p38 MAPKα and β that has been shown to prevent SOD1^G93A^-induced motor neuron death in vitro^13^. When tested at 2 μM, we found that SB-203580 was able to correct SOD1^G93A^-induced deficits in axonal retrograde transport in vitro (Fig. 5a). However, SB-203580 is also known to inhibit c-Jun N-terminal protein kinase 2/3 (JNK2/3)^28^, a pathway which has been shown to regulate survival in SOD1^E100G^ human induced pluripotent stem cell (hiPSC)-derived motor neurons^29^. Therefore, to demonstrate that inhibition of JNK2/3 is not involved in the restoration of axonal retrograde transport, we tested SB-239063, a second generation p38 MAPKα and β inhibitor with enhanced specificity^30^. Similarly to SB-203580, SB-239063 was able to rescue axonal transport in vitro in SOD1^G93A^ motor neuron cultures (Fig. 5b). Importantly, SB-239063 does not affect JNK activity in wild type and SOD1^G93A^ motor neurons (Fig. 5c, e), whereas p38 MAPK is strongly inhibited (Fig. 5d,f). This result is in line with our primary screen (Supplementary Fig. 2A**;** Supplementary Table 1) and previous results^30^ where JNK inhibition had no effect. Additionally, this compound reduced the hyperphosphorylation of neurofilament heavy chain (Supplementary Fig. 6A), which occurs in SOD1^G93A^ mice through increased p38 MAPK activity^16^.

**Fig. 6:**
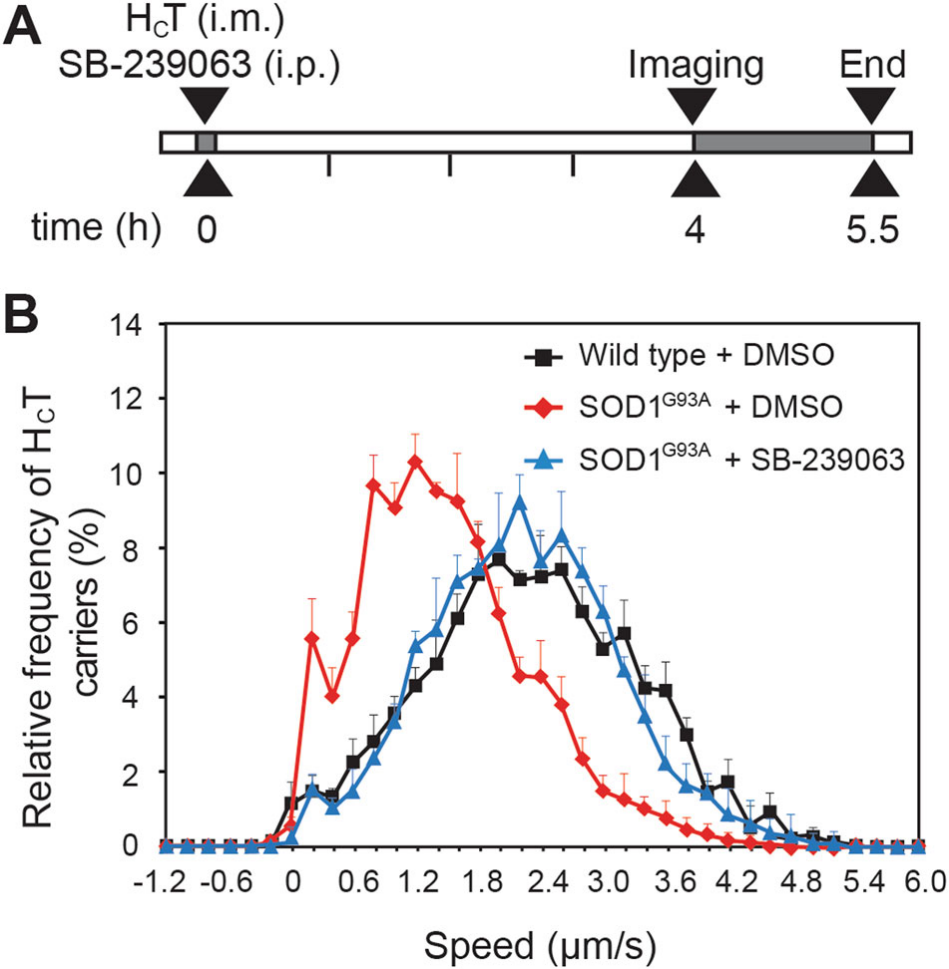
Inhibition of p38 MAPK correct axonal transport defects in vivo in SOD1^G93A^ mice. **a** Mice were anesthetised and AlexaFluor 555-conjugated H_C_T was injected intramuscularly (i.m.) into the tibialis anterior (TA) and gastrocnemius muscles (GC). At the same time, 100 mg/kg SB-239063 or vehicle control was administered via intraperitoneal injection (i.p.). The animals were allowed to recover for 4 h, then re-anesthetised and axonal transport imaged in the exposed sciatic nerve. **b** Axonal retrograde transport in single axons of the sciatic nerve was assessed in 73 d wild type (black triangles), SOD1^G93A^ (red diamonds) and SOD1^G93A^ mice treated with 100 mg/kg SB-239063 (blue triangles). SOD1^G93A^ mice displayed a significant impairment in axonal retrograde transport (*p* < 0.0001; Kruskal–Wallis test, followed by Dunn’s multiple comparison test) compared to wild type, whilst SOD1^G93A^ mice treated with SB-239063 showed transport speeds similar to those of wild-type mice (*p* = 0.5) (wild type: 161 carriers, n (animals) = 4; SOD1^G93A^: 185 carriers, *n* = 5; SOD1^G93A^ + 100 mg/kg SB-239063: 238 carriers, *n* = 4; data shown as mean ± SEM). Treatment of SOD1^G93A^ mice with vehicle was found to have no effect on in vivo axonal transport speeds

SB-239063 is also able to cross the blood brain barrier and pharmacokinetic analysis revealed that an intraperitoneal (i.p.) dose of 100 mg/kg allowed for circulating concentrations in the brain, spinal cord and muscle to reach, albeit transiently, levels similar to those used in our in vitro axonal transport assays (Supplementary Fig. 5B, C). Early symptomatic (73 ± 3 d) SOD1^G93A^ mice were treated with 100 mg/kg SB-239063 (i.p.) and axonal retrograde transport was assessed by imaging the sciatic nerve 4 h later in live, anaesthetised mice^31^ (Fig. 6a). This treatment was able to ameliorate the deficits in axonal retrograde transport of SOD1^G93A^ mice, restoring transport rates to those observed in wild-type animals (SOD1^G93A^ untreated versus SOD1^G93A^ + 100 mg/kg SB-239063: *p* < 0.0001; Fig. 6b). Similar results were obtained when  the concentration of SB-239063 was reduced to 10 mg/kg (data not shown).

### Effects of long-term treatment with SB-239063 on muscle physiology

**Fig. 7:**
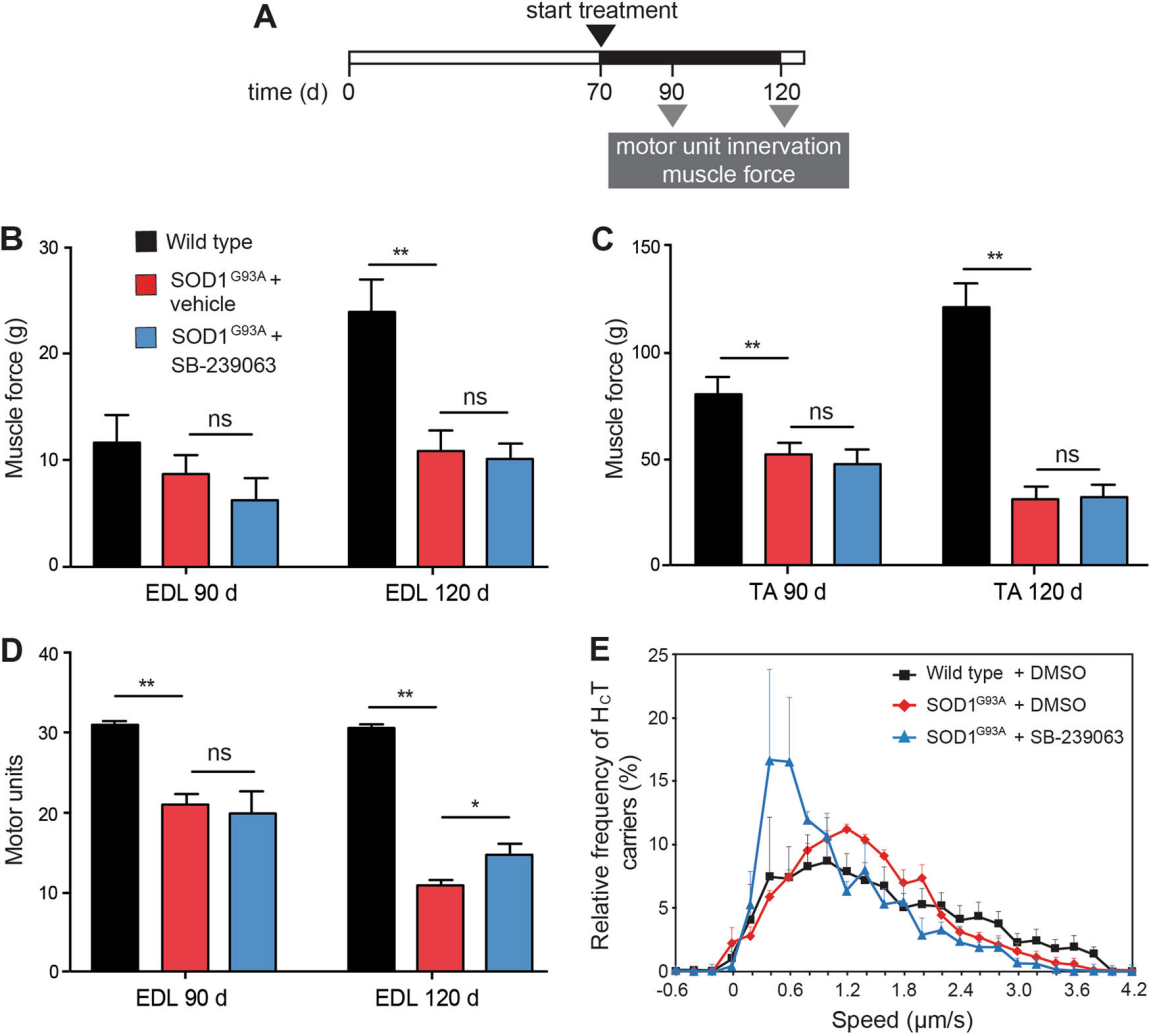
Effects of long-term treatment with SB-239063 on distal hindlimb muscle function and axonal transport. **a** Timeline of treatment of SOD1^G93A^ mice with SB-239063. Mice were injected i.p. with 10 mg/kg SB-239063 twice daily until the day of experimentss. **b** Maximum tetanic force by extensor digitorum longus (EDL) muscles in response to tetanic stimulation, measured at 90 and 120 d of age (minimum of five mice in each experimental group). Data shown as mean ± SEM. ** *p* < 0.01 (two-way ANOVA with Tukey’s multiple comparison test). **c** Maximum tetanic force measured in the TA muscles in response to tetanic stimulation, assessed at 90 and 120 d of age (minimum of six mice in each experimental group). Data shown as mean ± SEM. Ns, not significant; ** *p* < 0.01 (two-way ANOVA with Tukey’s multiple comparison test). **d** The number of motor units innervating the EDL muscle, assessed at 90 and 120 d (minimum of six mice in each experimental group). Treatment with SB-239063 induced a mild protection of EDL motor units at 120 d. Data shown as mean ± SEM. ** *p* < 0.01 (two-way ANOVA with Tukey’s multiple comparison test). **e** The axonal transport of AlexaFluor 555-conjugated H_C_T cargoes was measured in live anaesthetized mice at 90 d (wild type: 102 carriers n (animals) = 5; SOD1^G93A^ + vehicle: 98 carriers, *n* = 4; SOD1^G93A^ + 100 mg/kg SB-239063: 124 carriers, *n* = 4; data shown as mean ± SEM)

We next investigated whether long-term treatment with SB-239063 impacts on ALS progression. SOD1^G93A^ and wild-type littermates were treated with either 10 mg/kg SB-239063 or vehicle control (1% methylcellulose) i.p. twice daily until termination at 90 (symptomatic) and 120 d (end-stage of disease) (Fig. 7a). Hindlimb muscle function, including maximum tetanic tension in tibialis anterior (TA) and extensor digitorum longus (EDL) muscles, was determined in live anaesthetised animals at 90 and 120 d. In SOD1^G93A^ mice, TA and EDL muscles become progressively weaker, losing approximately 30% and 60% of muscle force at 90 and 120 d, respectively (Fig. 7b). Treatment with SB-239063 had no significant effect on force production of EDL or TA muscles (Fig. 7b, c).

The number of functional motor units innervating the EDL muscle in vehicle-treated SOD1^G93A^ mice is significantly reduced by 90 d (from 30.8 ± 0.6 in wild-type mice to 21.2 ± 0.8, *p*  < 0.0001; Fig. 7d), with a further loss occurring by 120 d (11.3 ± 0.7, *p*  < 0.0001; Fig. 7d). Whilst treatment with SB-236093 had no effect on motor unit survival at 90 d (20.0 ± 1.7 motor units; *p* = 0.79), a significant protection was observed at 120 d in SOD1^G93A^ mice treated with SB-239063 (14.7 ± 1.2 compared to 11.9 ± 2 functional motor units in SOD1^G93A^ mice treated with vehicle control; *p* = 0.0073; Fig. 7d).

We also examined whether hindlimb muscle innervation in SOD1^G93A^ mice was affected by long-term treatment with SB-239063 by assessing neuromuscular junctions (NMJs) in lumbrical muscles. These muscles innervate the digits of the hindlimbs and are one of the most distally affected muscle groups in SOD1^G93A^ mice^32^. Endplates were scored according to whether they were fully innervated (arrows), partially innervated (arrowheads), or denervated (double arrows; Supplementary Fig. 7A). At 90 d, more denervated endplates were found in SOD1^G93A^ (36.4 ± 8.9%) than wild-type mice (9.6 ± 1.6%; *p* = 0.071). By 120 d, 42.8 ± 2.8% of endplates were denervated in SOD1^G93A^ mice (*p* = 0.0062; Supplementary Fig. 7B). Treatment with SB-239063 had no significant effect on preserving muscle innervation in SOD1^G93A^ mice at both 90 and 12 d (Supplementary Fig. 7B).

These results show that, with the exception of a mild protection of EDL motor units at 120 d (Fig. 7d), long-term treatment with SB-239063 at an early symptomatic stage of disease did not lead to significant functional improvement in SOD1^G93A^ mice. However, when we assessed axonal transport in SOD1^G93A^ mice at 90 or 120 d, we found that axonal retrograde transport remained severely impaired despite the long-term treatment with SB-239063 (Fig. 7e and Supplementary Fig. 7C, D).

As shown previously^3^, symptomatic (90 d) and late symptomatic (120 d) SOD1^G93A^ mice display subtle deficits in axonal transport. This is likely due to the decrease in ALS-susceptible motor neuron axons observed at late time points of disease progression, and the increase in the contribution of ALS-resistant neurons to overall axonal transport rates. We therefore assessed the effects of long-term treatment with SB-239063 in pre-symptomatic 70 d old SOD1^G93A^ mice, the same age at which we found acute treatment to completely revert transport deficits (Fig. 6). 50 d old SOD1^G93A^ mice were treated with SB-239063 (10 mg/kg, twice daily) for 20 d (Supplementary Fig. 7E). Surprisingly, this treatment regime had very different effects than an acute treatment, since it failed to restore axonal transport in SOD1^G93A^ mice (Supplementary Fig. 7F). This is likely due to overt toxicity of long-term treatment with SB-239063, which caused sustained weight loss (Supplementary Fig. 7G**)**, and spleen and liver toxicity (Supplementary Fig. 7H). The methylcellulose used as vehicle is likely to have contributed to these side effects^33,34^. Additionally, the inability to maintain effective concentrations of SB-239063 in the brain may have contributed to the lack of long-term improvement of the SOD1^G93A^ ALS phenotype.

Altogether, these results demonstrate that genetic and acute pharmacological inhibition of p38 MAPKα is sufficient to reverse the axonal retrograde transport deficits observed in motor neurons of SOD1^G93A^ mice both in vitro and in vivo. However, the pharmacology and formulation of SB-239063 was suboptimal in our experimental design. As a result, long-term treatment with this p38 MAPK inhibitor, at least in 1% methylcellulose, has significant toxic side effects and, with the exception of a mild protection of EDL motor units, consequently failed to improve either axonal transport or muscle function in SOD1^G93A^ mice.

## Discussion

Deficits in axonal transport have been implicated in the pathogenesis of several neurodegenerative diseases. In ALS, defects in both anterograde^2,3,12^ and retrograde^2,3,24^ axonal transport have been observed at early stages of disease. This suggests that abnormalities in this pathway may play a role in the initiation and/or early progression of ALS. However, to directly demonstrate a role for axonal transport deficits in triggering neurodegeneration, it is necessary to show that restoring axonal transport alters disease progression. The findings reported in this study identify pharmacological inhibitors of p38 MAPKα that are capable of reversing the transport deficits observed in SOD1^G93A^ motor neurons and thereby provide a valuable opportunity to establish whether ameliorating transport deficits modifies disease progression in ALS. However, we were unable to conclusively test this hypothesis because long-term in vivo treatment of SOD1^G93A^ mice with SB-239063 in 1% methylcellulose i.p caused overt liver and spleen toxicity.

Although defects in axonal transport have been previously shown in SOD1^G93A^ motor neurons^3,24^, the molecular mechanism responsible for these deficits is currently unknown. Since several kinases have been linked to axonal transport regulation^35^ and kinase overactivation has been suggested to be a pathological hallmark of ALS^35,36^, we screened a library of kinase inhibitors for enhancers of axonal retrograde transport in motor neurons. We found that p38 MAPK inhibitors were able to completely restore axonal transport of signalling endosomes in primary SOD1^G93A^ motor neurons (Fig. 3), whilst having no effect on transport speeds in wild-type cells (Supplementary Fig. 2**)**. These results suggest that SOD1^G93A^ causes the sustained activation of the p38 MAPK signalling cascade (Fig. 3 and (Supplementary Fig. 4), which drives the pathological effects of mutant SOD1 on axonal transport. Interestingly, we observed a disease-dependent increase in p38 MAPK activation in SOD1^G93A^, but not in SOD1^WT^ mice (Fig. 3 and (Supplementary Fig. 4), which correlated with the appearance and severity of axonal retrograde transport defects^3^. Supporting this evidence, other groups have previously demonstrated activation of p38 MAPK in spinal cords of both familial and sporadic ALS patients^14^ and in SOD1^E100G^ hiPSC-derived motor neurons^29^.

p38 MAPKα inhibits axonal anterograde transport through direct phosphorylation of kinesin-1^12^, thus reducing the ability of the motor to move along axonal microtubules. In our study, we found that p38 MAPKα inhibitors restore deficits in axonal retrograde transport, suggesting a model in which one or more subunits of cytoplasmic dynein or motor adaptors, are regulated by phosphorylation^35,37,38^, as previously reported for huntingtin^39^ and Ndel1^40^.

The p38 MAPK-mediated inhibition of axonal transport may also be indirect. p38 MAPKα phosphorylates the medium and heavy neurofilament subunits (NFM and NFH), and colocalises with phosphorylated neurofilaments in SOD1^G93A^ motor neurons^11^. Aberrant hyperphosphorylation of neurofilaments has been proposed to slow their transport and induce bundling^41^, which in turn may lead to the deficits of axonal transport observed in ALS. Furthermore, tau is a known substrate of p38 MAPK and has been shown to be hyperphosphorylated at a pre-symptomatic stage in SOD1^G37R^ mice^42^. Phosphorylation of tau by p38 MAPK decreases its association with microtubules^43^, which was proposed to induce microtubule destabilisation and disruption of axonal transport. In our study, treatment with SB-239063 decreases NFH hyperphosphorylation, thus demonstrating target engagement in vitro. However, since this effect was only observed after 24 h of treatment (Supplemental Fig. 6), it may not be the main mechanism underlying the effects of SB-239063 on axonal transport.

Although some controversy exists regarding the role of axonal transport deficits in ALS^8,44^, a large body of evidence indicates that restoring axonal transport could be a promising therapeutic strategy. For example, pharmacological inhibition or knockout of histone deacetylase 6 (HDAC6) accelerates axonal transport in motor and sensory neurons^45–47^, causing an improvement in motor behaviour in a peripheral neuropathy model^47^, and increasing motor neuron survival and lifespan in SOD1^G93A^ mice^48^. In addition, the p38 MAPKα and β inhibitor SB-203580, shown in this study to restore axonal transport, reduces SOD1^G93A^ motor neuron death in vitro^13^.

A p38 MAPK inhibitor used in this study, SB-239063, is potentially suitable for in vivo use, since it is able to cross the blood brain barrier (Supplementary Fig. 6) and restore axonal transport in SOD1^G93A^ mice (Fig. 6). In principle, SB-239063 therefore holds potential to assess whether axonal transport defects play a significant role in the pathogenesis of ALS. However, optimisation of alternative formulations and/or treatment regimes are necessary to overcome the general toxicity found upon i.p administration. Although transgenic mice overexpressing human SOD1^G93A^ are the best-characterised model of ALS to date^49^, there are now several familial ALS models available^50,51^. Validating our results in other ALS models will allow us to determine whether inhibition of p38 MAPKα represents a general therapeutic strategy for ALS or if p38 MAPK overactivation is a pathological mechanism specific for mutant SOD1.

Deficits in axonal transport have been shown to be a hallmark of other neurodegenerative diseases^2,7,52^, including Alzheimer’s disease (AD) and hereditary neuropathies. Henceforth, the identification of novel enhancers of axonal transport could yield novel therapeutic approaches for these presently untreatable pathologies. Interestingly, the p38 MAPKα selective inhibitor MW01-18-150SRM has been found to attenuate disease progression in two AD mouse models^53^, suggesting that pathological mechanisms may be shared among distinct neurodegenerative diseases.

## Materials and methods

### Animals and tissue collection

All experiments were carried out following the guidelines of the UCL-Institute of Neurology Genetic Manipulation and Ethic Committees and in accordance with the European Community Council Directive of November 24, 1986 (86/609/EEC). Animal experiments were undertaken under license from the UK Home Office in accordance with the Animals (Scientific Procedures) Act 1986 (Amended Regulations 2012) and the GSK Policy on the Care, Welfare and Treatment of Animals. Female transgenic mice carrying a human wild-type SOD1 (B6SJLTg[SOD1]2Gur/J) or mutant SOD1^G93A^ (TgN[SOD1-G93A]1Gur) gene were used in these experiments. Colonies were maintained by breeding male heterozygous carriers with female (C57BL/6 × SJL) F1 hybrids. Mice were genotyped for the human SOD1 transgene from ear or tail genomic DNA. Spinal cords were harvested from terminally anaesthetized mice at different stages of disease progression, snap-frozen in liquid nitrogen and stored at −80 °C.

Female mutant SOD1^G93A^ mice at different ages (25 or 70 d) were randomly allocated into vehicle treated (1% methylcellulose; Sigma 274429, St Louis, MO) and SB-239063 (GlaxoSmithKline, Singapore, SG) treated groups. A separate group of wild-type mice was also included, which was treated with vehicle. Mice were injected intraperitoneally twice daily with 10 mg/kg SB-239063 until the day of experiment. In each experimental group and assessment, 5–8 mice were included.

### Motor neuron cultures

ES cell-derived motor neurons were cultured as previously described^17^. Primary motor neurons were isolated from spinal cords of E12.5–13.5 mouse embryos^54^. Briefly, embryos were sacrificed, the spinal cords dissected and the ventral horns isolated and dissociated by incubation with trypsin, followed by incubation with DNase and centrifugation through a BSA cushion. The resulting cell pellet was resuspended in complete motor neuron medium [Neurobasal (ThermoFisher, Waltam, MA), 2% v/v B27 supplement (ThermoFisher), 2% heat inactivated horse serum, 1% GlutaMAX (ThermoFisher), 25 μM β-mercaptoethanol, 10 mg/ml recombinant rat CNTF (R&D Systems, Minneapolis, MN), 100 pg/ml recombinant rat GDNF (R&D Systems), 1 ng/ml recombinant human BDNF (R&D Systems) and Pen/Strep antibiotics]. Cells were immediately plated on poly-DL-ornithine/laminin coated plates and maintained in culture for 5–8 d (5–8 DIV).

### Accumulation and in vitro axonal retrograde transport assays

The accumulation of AlexaFluor 555-conjugated H_C_T and α-p75^NTR^ in the cell body of motor neurons was performed as previously described^17^, except that the H_C_T and α-p75^NTR^ were applied in complete medium. Internalised probes were quantified using the Cell Profiler software^17,18^. For drug treatments, compounds were dissolved in dimethylsulphoxide (DMSO) and were applied at the same time as the H_C_T and α-p75^NTR^.

Axonal retrograde transport assays with AlexaFluor 647-conjugated H_C_T and α-p75^NTR^ were performed as previously described^55^ and quantified using Motion Analysis software (Kinetic Imaging, Nottingham, UK). Compounds were applied at the same time as H_C_T or α-p75^NTR,^ and the speed distribution profiles of their carriers have been obtained using a 0.2 μm/s binning interval^3^.

### Reagents and antibodies

Chemicals were from Sigma-Aldrich, unless otherwise stated. SB-239272, SB-239063 and SB-203580 were from GlaxoSmithKline; ciliobrevin A was from InterBioScreen (Chernogolovka, RU). Tissue culture media and supplements were purchased from Life Technologies. Antibodies for phospho-p38 MAPK (Thr180/Tyr182; #4511), p38 MAPKα (#9211), p38 MAPKγ (#2307), p38 MAPKδ (#2308), pan-p38 MAPK (#9212) and JNK (#9252) were from Cell Signalling (Danvers, MA). p38 MAPKβ (PA1-41154) was from ThermoFisher and phospho-JNK (g-7; #sc-6254) was from Santa Cruz Biotechnology (Dallas, TX). The GAPDH antibody (mab374) was from Merck Millipore (Billerica, MA). The mouse monoclonal antibody specific for phosphorylated neurofilament heavy chain (SMI34; #835503) was from Biolegend (San Diego, CA). The antibody for GFP (4E12/8) was a kind gift from the Cancer Research UK London Research Institute monoclonal facility. The polyclonal antibody against the extracellular domain of p75^NTR^ (α-p75^NTR^; 5411) used in this study has been previously described^5^.

### Lentivirus preparation and transduction

shRNA constructs directed against p38 MAPKα (MSH030695-LVRU6GP) and δ (MSH0306954-LVRU6GP) and scrambled controls were purchased from the OmicsLink™ shRNA clone collection (GeneCopoeia, Rockville, MD). All constructs were in psi-LVRU6GP plasmids with an eGFP reporter gene. Briefly, Lenti-X 293 T cells (ClonTech, Mountain View, CA) were co-transfected with shRNA, packaging, and envelope plasmid vectors using Lipofectamine 3000 (ThermoFisher). Medium containing lentiviral particles was collected every day for 3 d after transfection. Medium containing viral particles was concentrated using LentiX Concentrator (ClonTech) and the lentiviral particles resuspended in complete motor neuron medium. The lentivirus titre was determined using the Lenti-X p24 Rapid Titre kit (ClonTech) and lentiviral particles were stored at −80 °C until further use. To transduce motor neurons, lentivirus was added to the culture medium 6 h after plating. The medium was replaced 16 h after transduction, and motor neurons were assayed one week later.

### Western blotting

Motor neuron and spinal cord lysates were prepared in RIPA buffer (50 mM Tris-HCl pH 7.5, 150 mM NaCl, 1% NP-40, 0.5% sodium deoxycholate, 0.1% SDS, 1 mM EDTA, 1 mM EGTA) containing Halt™ phosphatase and protease inhibitor cocktail (1 in 100; ThermoFisher) and let to incubate on ice for at least 30 min. Lysates were centrifuged at 14,800 rpm for 15 min at 4 °C and protein concentration determined using Pierce™ BCA Protein Assay (ThermoFisher). Samples (20 µg) were run on 4–12% NuPAGE Bis-Tris gradient gels (ThermoFisher) and proteins blotted onto polyvinylidene fluoride (PVDF) membranes (Merck Millipore). Membranes were blocked in 5% bovine serum albumin (BSA) dissolved in Tris-buffered saline (TBS) containing 0.5% Tween-20 (TBST) for 1 h at 4 °C and then incubated with primary antibodies overnight at 4 °C. Blots were washed and incubated with appropriate horseradish peroxidase (HRP)-conjugated secondary antibodies (GE Healthcare, Little Chalfont, UK). Immunoreactivity was detected using Crescendo ECL substrates (Merck Millipore) and FujiFilm X-ray film (ThermoFisher). Quantification was performed using ImageJ. For the analysis of neurofilament phosphorylation, motor neurons (7 DIV) were treated for either 2 h or 24 h with 2 µM SB-239063 or vehicle control (DMSO). Five micrograms of protein lysates were run on a 7.5% acrylamide gels, blotted onto PVDF membranes and then stained with a mouse monoclonal specific for phosphorylated NFH (SMI34).

### Immunofluorescence

Motor neurons were fixed in 4% paraformaldehyde in phosphate buffered saline (PBS) for 15 min at room temperature, permeabilized with 0.1% Triton X-100 in 5% BSA in PBS for 60 min and then incubated with the relevant primary antibodies in blocking solution (5% BSA in PBS) overnight at 4 °C. Motor neurons were washed in blocking solution and then incubated with the appropriate secondary antibodies (1:400 in blocking solution). Cells were washed in PBS, mounted in Mowiol 4–88 and imaged using a Zeiss LSM 780 confocal microscope equipped with a 63 × oil-immersion objective (Zeiss, Oberkochen, DE).

### In vivo axonal retrograde transport assays

In vivo axonal transport assays were performed as previously described^3,31^. Briefly, mice were anesthetized with isoflurane (National Veterinary Services, Stoke-on-Trent, UK), and AlexaFluor 555-conjugated H_C_T (13 μg) and BDNF (50 ng) were injected intramuscularly into the exposed TA and gastrocnemius (GC) muscles of one hind leg, and the wound sutured. Mice were then left to recover and kept under standard conditions with unlimited food and water supply. Four hours later, animals were re-anesthetized and the sciatic nerve exposed. Mice were placed on a heated stage in an environmental chamber (both kept at 37 °C) and axonal transport was imaged in the intact sciatic nerve by time-lapse confocal microscopy. Images of axons were acquired every 3–4 s with an inverted Zeiss LSM 780 equipped with a 63 × oil-immersion objective (Zeiss, Oberkochen, DE). SB-239063 was suspended in a solution of 1% methylcellulose and delivered by intraperitoneal injection at the same time as the AlexaFluor555-conjugated H_C_T was injected intramuscularly.

### Assessment of muscle force, number of motor units and endplate innervation

Experimental animals were anesthetised using isoflurane on the day of assessment. The distal tendon of the TA and EDL muscles was dissected and connected to a force transducer, and the exposed sciatic nerve was attached to a stimulating electrode. Maximum tetanic force as well as the number of functional motor units were assessed as previously described^56^.

At the end of the axonal transport experiments, animals were euthanized and the lumbrical muscles were removed from the hind paws, fixed in 4 % paraformaldehyde for 5 min and then stained with a combination of antibodies against neurofilament (2HG3) and SV2 (both from Developmental Studies Hybridoma Bank, Iowa City, IA) as well as with AlexaFluor 568-labelled bungarotoxin (BTX; ThermoFisher)^57^. Endplate occupancy was scored according to the level of co-localisation between the endplate (visualized by BTX labelling) and terminal motor axon (combined neurofilament and SV2 labelling). Endplates were classified into three categories: fully innervated, partially innervated or denervated, according to the overlap of endplate labelling with the neurofilament/SV2 staining. For each animal a total of minimum 100 endplates were analysed from different regions of the muscle to include endplates innervated by multiple terminal motor axons. Innervation was then expressed as a percentage of total number of endplates assessed.

### Statistical analysis

Statistical analysis was performed using Graphpad Prism software (La Jolla, CA). Unless otherwise stated, data is expressed as mean ± SEM. To determine the most appropriate statistical test to use, data were tested for normality using three tests: D’Agostino & Person omnibus normality test, Shapiro-Wilk normality test and KS normality test. If the data were found to be normally distributed, either a Student’s t-test (*n* = 2 groups to compare) or one-way/two-way analysis of variance (ANOVA) (*n* > 2 groups to compare), followed by Dunnett’s or Sidak’s multiple comparisons test, was used. Muscle physiology data were analysed using a two-way ANOVA with Tukey’s multiple comparisons test. If the data were not found to be normally distributed, a Kruskal–Wallis test was used, followed by Dunn’s multiple comparison test (*n* > 2 groups to compare). If there were too few data points to accurately test for normality, data were assumed to be normally distributed. The test used and associated *p* values are indicated in the figure legends.

## Electronic supplementary material

Supplemental Material
