# Supplementary material for: Inhibiting p38 MAPK alpha rescues axonal retrograde transport defects in a mouse model of ALS
Source: Cell Death Dis. 2018 May 22;9(6):596. doi: 10.1038/s41419-018-0624-8 (PMC5964181; doi:10.1038/s41419-018-0624-8)
Supplement: Supplementary file 1 — Supplemental Material [file 41419_2018_624_MOESM1_ESM.pdf]

Supplementary figure legends

Supplementary Figure 1. Modulation of axonal retrograde transport is detected by the accumulation assay. (A-D) Treatment of ES cell-derived motor neurons with 1 mM EHNA causes a significant decrease in the accumulation of H_CT (A) and α-p75^{NTR} (C) in the cell body. ES cell-derived motor neurons were incubated with AlexaFluor 555-conjugated H_CT or α-p75^{NTR} and either DMSO or 1 mM EHNA for 2 h. Cells were then acid-washed, fixed, permeabilised and stained for α-p75^{NTR}. The amount of intracellular H_CT (B) and α-p75^{NTR} (D) was quantified as the mean staining intensity per pixel in the cell body. (**E-F**) Defective transport in SOD1^{G93A} motor neurons is observed as a decrease in accumulation of H_CT in the cell body. Primary motor neurons from E13 SOD1^{G93A} embryos were incubated with AlexaFluor 555-conjugated H_CT for 2 h. Cells were then acid-washed and fixed. The amount of intracellular H_CT (F) was quantified as the mean staining intensity per pixel in the cell body. Results are expressed as a percentage of the control (A-D) or wild type (E-F) ($n \geq 25$ cell bodies per condition; $n = 3$ independent experiments). *** $p< 0.001$, **** $p< 0.0001$ (unpaired Student's *t* test). Scale bars, 10 μm.

Supplementary Figure 2. Compound A1 accelerates axonal transport in SOD1^{G93A} but not in wild type motor neurons. (**A**) The results of a small molecule chemical screen are shown as an XY plot of the normalized mean staining intensity for α-p75^{NTR} versus AlexaFluor 555-conjugated H_CT. α-p75^{NTR} was detected as described in Figure 2. The red dot highlights the lack of effect of a reported JNK inhibitor in this assay. This result is in line with data shown in

Figure 5. The yellow rectangle corresponds to three times the standard deviation of the dataset. The negative control (EHNA) is shown in blue (n ≥ 25 cell bodies analysed per condition; n = 3 repeats). (**B**) Speed profiles of H_CT carriers in wild type motor neurons treated with DMSO (black squares) or 2 μM A1 (light blue triangles). Comparison of the curves reveals that compound A1 has no effect on the speed of axonal retrograde transport in wild type neurons (wild type: 94 carriers, 13 axons; wild type + 2 μM A1: 108 carriers, 11 axons; 4 independent experiments).

Supplementary Figure 3. Target validation of the small molecule screen. (**A**) Summary of the results of the small molecule chemical screen plotted as the normalized mean staining intensity values for α-p75^{NTR} versus H_CT. An additional fourteen p38 MAPK inhibitors were screened for their effect on the accumulation of H_CT and α-p75^{NTR}. Active compounds were defined as those able to increase the accumulation of α-p75^{NTR} and H_CT by at least three times the standard deviation of the entire dataset (represented by the yellow rectangle); n ≥ 25 cell bodies per condition, n = 3 repeats per conditions. The active compounds are shown in red, whilst the negative control (EHNA) is shown in blue. (**B-E**) Speed profiles of AlexaFluor 555-conjugated H_CT carriers in SOD1^{G93A} motor neurons treated with 2 μM of each of the active compounds (wild type: 74 carriers, 8 axons; SOD1^{G93A}: 119 carriers, 7 axons; SOD1^{G93A} + 2 μM A3: 120 carriers, 7 axons (B); SOD1^{G93A} + 2 μM C3: 117 carriers, 8 axons (C); SOD1^{G93A} + 2 μM D3: 166 carriers, 9 axons (D); SOD1^{G93A} + 2 μM H2: 94 carriers, 7 axons; 3 independent experiments (E)).

Supplementary Figure 4. p38 MAPK is activated in both embryonic motor neurons and adult SOD1^{G93A} spinal cord. Western blot showing levels of phospho-p38 MAPK in spinal cord lysates from wild type, SOD1^{G93A} and SOD1^{WT} overexpressing mice *(top)*. Active p38 MAPK was detected with a phosphospecific pT180/pY182 α-p38 MAPK antibody, whilst the total content of p38 MAPK was assessed by a pan α-p38 MAPK antibody. The ratios of p-p38 MAPK / total p38 MAPKα signals are shown in the lower part of the panel. The ratios of mutant and wild type hSOD1 at different ages were been normalised to the ratio of age matched wild type littermates (taken as 1). p38 MAPK shows increased activation in pre-symptomatic (36 d) and early symptomatic (73 d) SOD1^{G93A} mice. Spinal cords from SOD1^{WT} overexpressing mice show no change in p38 MAPK activation compared to wild type controls (n = 1 animal per condition).

Supplementary Figure 5. Lentiviral-mediated transduction of primary motor neurons. (**A**) Western blot showing lentivirus-mediated knockdown of p38 MAPKα in primary motor neurons (n = 1 experiment). (**B**) Quantification of western blots shows a reduction in p38 MAPKα levels with p38 MAPKα-targeted shRNA, whilst scrambled shRNA has no effect. (**C**) Example of western blot showing the lentivirus-mediated knockdown of p38 MAPKδ (*left*) and its quantification showing dose-dependent reduction of p38 MAPKδ expression (*right*). (**D**) Speed profiles of H_CT carriers in SOD1^{G93A} motor neurons transduced with scrambled shRNA (scram; red diamonds) and SOD1^{G93A} motor neurons transduced with p38 MAPKδ shRNA (brown triangles). Comparison of the curves reveals that knockdown of p38 MAPKδ has no effect on axonal transport speeds

(SOD1^{G93A} + scrambled shRNA: 46 carriers, 10 axons; SOD1^{G93A} + p38 MAPKδ shRNA: 101 carriers, 12 axons; 3 independent experiments).

Supplementary Figure 6. SB-239063 affects neurofilament phosphorylation in primary motor neurons. (**A**) *Top panel*: Effect of 2 μM SB-239063 on the phosphorylation of neurofilament heavy chain detected by the SMI34 antibody in motor neurons isolated from wild type and SOD1^{G93A} mice. Actin was used as loading control (n = 3 independent experiments). *Lower panel*: western blot quantification revealed a reduction in the SMI34 signal in extracts from SOD1^{G93A} cultures after 24 h of treatment (n = 3 independent experiments). Data shown as mean ± SEM. ** $p< 0.01$ (two-way ANOVA followed by Tukey's comparison test). (**B**) Wild type mice were administered either 30 mg/kg or 100 mg/kg SB-239063 i.p. The free concentration in the brain, spinal cord and muscle was determined after 1 h. ^ based on fraction unbound in rat brain. ^^ based on fraction unbound in rat blood. (**C**) Predicted brain free concentration profiles upon administration of 30 mg/kg and 100 mg/kg of SB-239063 via i.p. at 0 and 8 h.

Supplementary Figure 7. Chronic treatment with SB-239063 does not restore axonal transport deficits in 70 day old SOD1^{G93A} mice and has severe side effects. (**A**) Muscle endplate occupancy in lumbricals muscles of the distal hindlimb. Examples show fully innervated endplates, with neurofilament heavy chain and synaptic vesicle protein 2 (NF/SV2, in green) labelling covering the endplate region (labelled by α-bungarotoxin (BTx) in red), indicating fully innervated endplates (arrows). Some endplates only

display partial neurofilament coverage, indicating these endplates are partially innervated (arrowheads), whereas in some cases endplates do not show any labelling for neurofilament/SV2, an indication for denervated endplates (double arrows). Scale bars, 20 μm. (**B**) Muscle endplate occupancy in lumbricals muscles of the distal hindlimb was assessed in wild type, vehicle-treated and SB-239063 treated $SOD1^{G93A}$ mice at 90 d (*left*) and 120 d (*right*) of age. Endplate occupancy was measured as the percentage of the total number of endplates assessed (n = minimum of 3 mice in each experimental group). The level of denervation was compared between each experimental group using a 2 way ANOVA with Tukeys multiple comparisons test. At 90 d there was significant denervation in $SOD1^{G93A}$ mice compared to controls (p=0.0264), which was also present at a later stage (120 d; p=0.0044). Treatment with SB-239063 did not result in a significant protection from denervation compared to vehicle-treated $SOD1^{G93A}$ mice (p=0.9998 at 90 d; p=0.8934 at 120 d). (**C**) Timeline of treatment of $SOD1^{G93A}$ mice with SB-239063. Mice were injected i.p. with 10 mg/kg SB-239063 twice daily until the day of experiment. (**D**) The axonal transport of AlexaFluor 555-conjugated H_CT cargoes was measured in live anaesthetized mice at 120 d (wild type: 128 carriers, n (animals) = 5; $SOD1^{G93A}$ + vehicle: 110 carriers, n = 4; $SOD1^{G93A}$ + 100 mg/kg SB-239063: 139 carriers, n = 5; data shown as mean ± SEM). (**E**) Timeline of treatment of $SOD1^{G93A}$ mice with SB-239063. Mice were injected i.p. with 10 mg/kg SB-239063 twice daily until the day of experiments. (**F**) Axonal transport in $SOD1^{G93A}$ mice at 70 d is not improved by long-term treatment with SB-239063 starting at 50 d (wild type: 187 carriers, n (animals) = 5; $SOD1^{G93A}$ + vehicle: 103 carriers, n = 3; $SOD1^{G93A}$ + 10 mg/kg SB-239063:

136 carriers, n = 4; data shown as mean ± SEM). (**G**) SOD1^{G93A} mice treated chronically, twice daily, with SB-239063 go through an initial phase of weight loss, compared to vehicle treated SOD1^{G93A} mice (wild type: n (animals) = 8; SOD1^{G93A} + vehicle: n = 9; SOD1^{G93A} + 10 mg/kg SB-239063: n = 7; data shown as mean ± SEM). (**H**) Chronic treatment with either vehicle or SB-239063 leads to severe spleno- and hepato-megaly in SOD1^{G93A} mice. Grid size: 10 mm.

Supplementary Table 1. Library of kinase inhibitors used for the primary screen. Selected active compounds are highlighted in grey.

Reference code	Compound name	Primary Target
2A1	GW693481X	TGFBR
2A2	GW572738X	JNK
3A1	GSK200398A	EGFR/ErbB2
3A2	GW651576X	EGFR/ErbB2
A1	SB-239272	p38 MAPK
A2	GSK248233A	RHO
A3	GW296115X	PDGFR
A4	GW589961A	TIE2/VEGFR2
A5	GW806742X	VEGFR
A6	GSK586581A	IKK
A7	GW410563A	VEGFR
B1	GW801372X	GSK3
B2	SB-772077-B	RHO
B3	SB-400868-A	TGFBR
B4	SB-220025-A	p38 MAPK
B5	GI261520A	EGFR/ErbB2
B6	GW824645A	CDK2
B7	GSK1023156A	PLK
C1	GW679410X	TGFBR
C2	SB-675259-M	GSK3
C3	GW440139A	RET
C4	GW820759X	p38 MAPK
C5	GW642125X	TIE2/VEGFR2
C6	GW275616X	TRKA
C7	GW406731X	RAF
D1	GW549390X	VEGFR
D2	GW561436X	p38 MAPK
D3	GW768505A	TIE2/VEGFR2
D4	GW856804X	TIE2/VEGFR2
D5	GW461104A	EGFR/ErbB2
D6	GSK180736A	RHO
D7	GW559768X	RET
E1	GW575808A	LCK
E2	GSK319347A	IKK
E3	GW743024X	p38 MAPK
E5	GSK1000163A	AKT
E6	GSK237700A	PLK
F1	GW632580X	CSF
F2	GW837331X	PLK
F3	SB-358518	GSK3
F4	GW275944X	CDK2
F5	GSK554170A	AKT
F6	GW513184X	GSK3

G1	GSK317354A	RHO
G2	GW284372X	EGFR/ErbB2
G3	SB-437013	TIE2
G4	GW305074X	RAF
G5	SB-737198	MSK
G6	GSK1173862A	IGF-1R
H1	GW819230X	GSK3
H2	SB-744941	MSK
H3	SB-725317	GSK3
H4	GSK182497A	EGFR/ErbB2
H5	GR105659X	TRKA
H6	GSK953913A	IKK

Supplementary Table 2. Library of kinase inhibitors used for the validation (secondary) screen. Selected active compounds are highlighted in grey.

Reference code	Compound name	Primary Target
A1	SB-226879	p38 MAPK
A2	GW775608X	p38 MAPK
A3	GW796921X	p38 MAPK
B1	SB-221466	p38 MAPK
B2	GW581744X	p38 MAPK
C2	GW607117X	p38 MAPK
C3	SB-223133	p38 MAPK
D1	GW618013A	p38 MAPK
D2	SB-242719	p38 MAPK
D3	GW434756X	p38 MAPK
E2	GW734508X	p38 MAPK
G1	GW769076X	p38 MAPK
H1	GW569293E	p38 MAPK
H2	GW782907X	p38 MAPK

Supplementary Table 3. Active p38 MAPKα compounds used *in vitro* and/or *in vivo* in this study.

Compound name	Primary Target	ChEMBL[♯]	References
SB-239063	p38 MAPKα	97162	(8, 9)
SB-203580	p38 MAPKα	10	(8)
SB-239272 (A1)	p38 MAPKα	275798	(8)
SB-223133	p38 MAPKα	1888586	(8)
GW796921X	p38 MAPKα	517666	(10)
GW434756X	p38 MAPKα	485745	(11)
GW782907X	p38 MAPKα	477978	(10)

[♯]https://www.ebi.ac.uk/chembl/

Figure S1

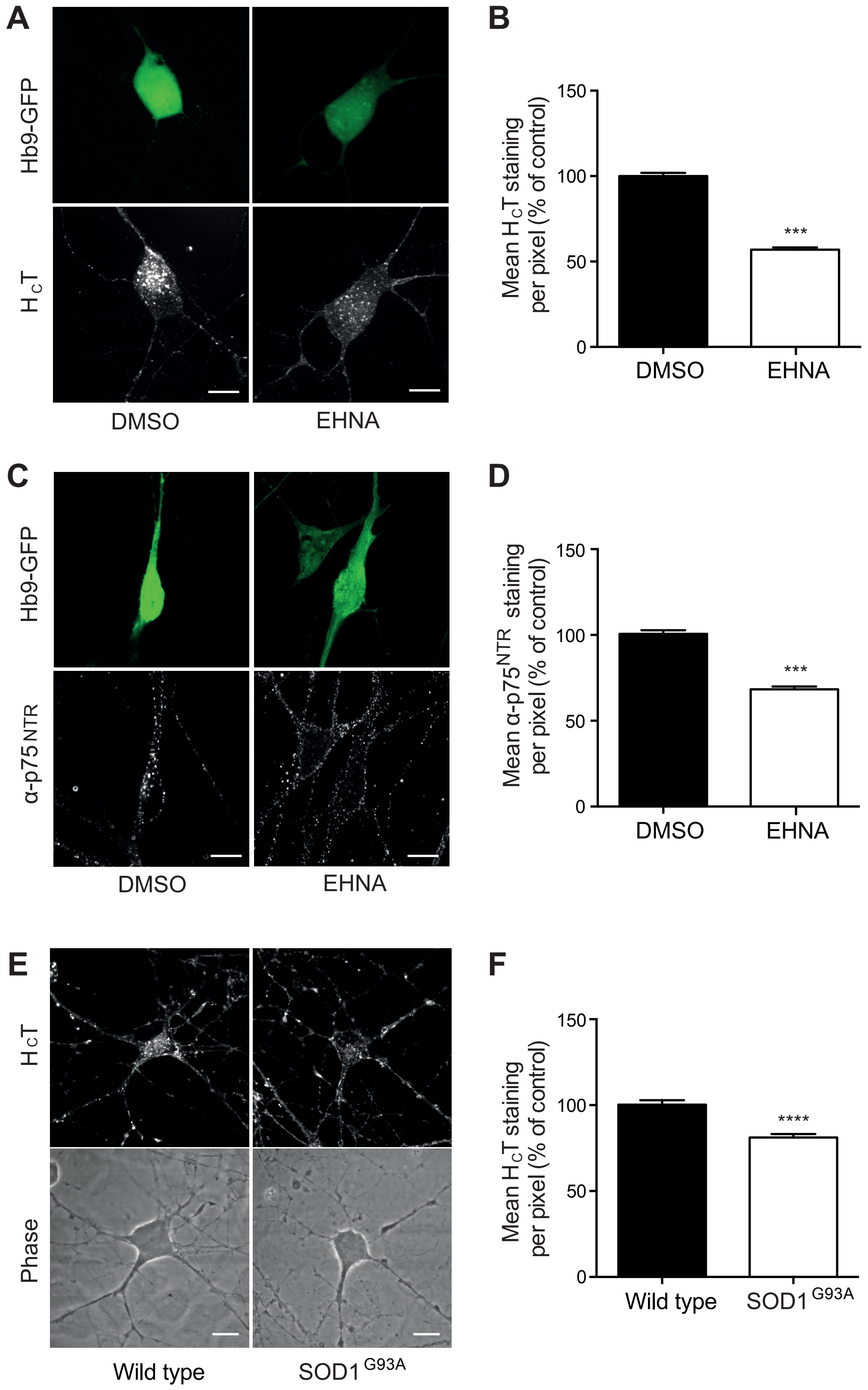

A
Hb9-GFP
H_CT
DMSO
EHNA
B
Mean H_CT staining per pixel (% of control)
150
100
50
0
***
DMSO
EHNA
C
Hb9-GFP
α-p75 NTR
DMSO
EHNA
D
Mean α-p75^{NTR} staining per pixel (% of control)
150
100
50
0
***
DMSO
EHNA
E
H_CT
Phase
Wild type
SOD1^{G93A}
F
Mean H_CT staining per pixel (% of control)
150
100
50
0
****
Wild type
SOD1^{G93A}

Figure S2

A

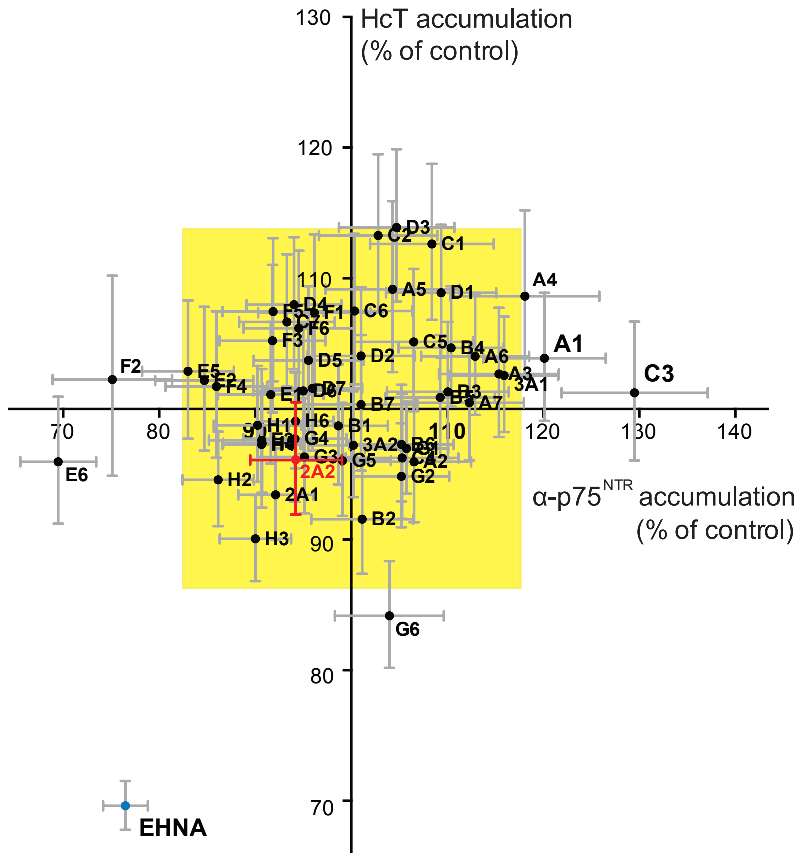
HcT accumulation
(% of control)
α-p75NTR accumulation
(% of control)
130
120
110
90
80
70
70
80
120
130
140
A1
C3
A4
D3
C2
C1
A5
D1
C6
D4
F1
F6
F3
C5
B4
A6
D2
D5
F2
E5
F4
A3
3A1
B3
A7
B7
H6
B1
3A2
G4
G3
G5
2A2
A2
G2
H2
2A1
B2
H3
E6
G6
EHNA

B

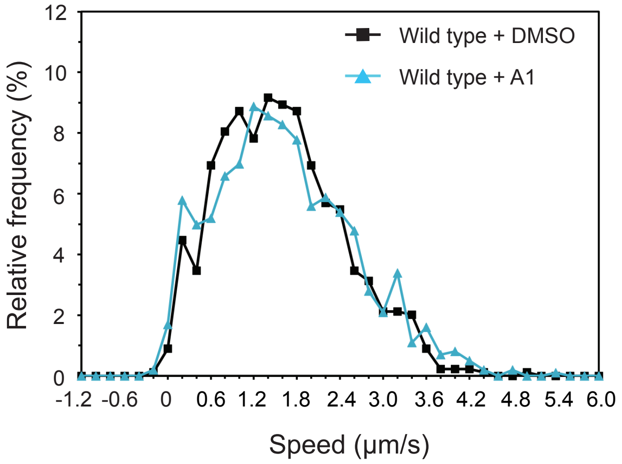
Wild type + DMSO
Wild type + A1
Relative frequency (%)
Speed (μm/s)
12
10
8
6
4
2
0
-1.2
-0.6
0
0.6
1.2
1.8
2.4
3.0
3.6
4.2
4.8
5.4
6.0

Figure S3

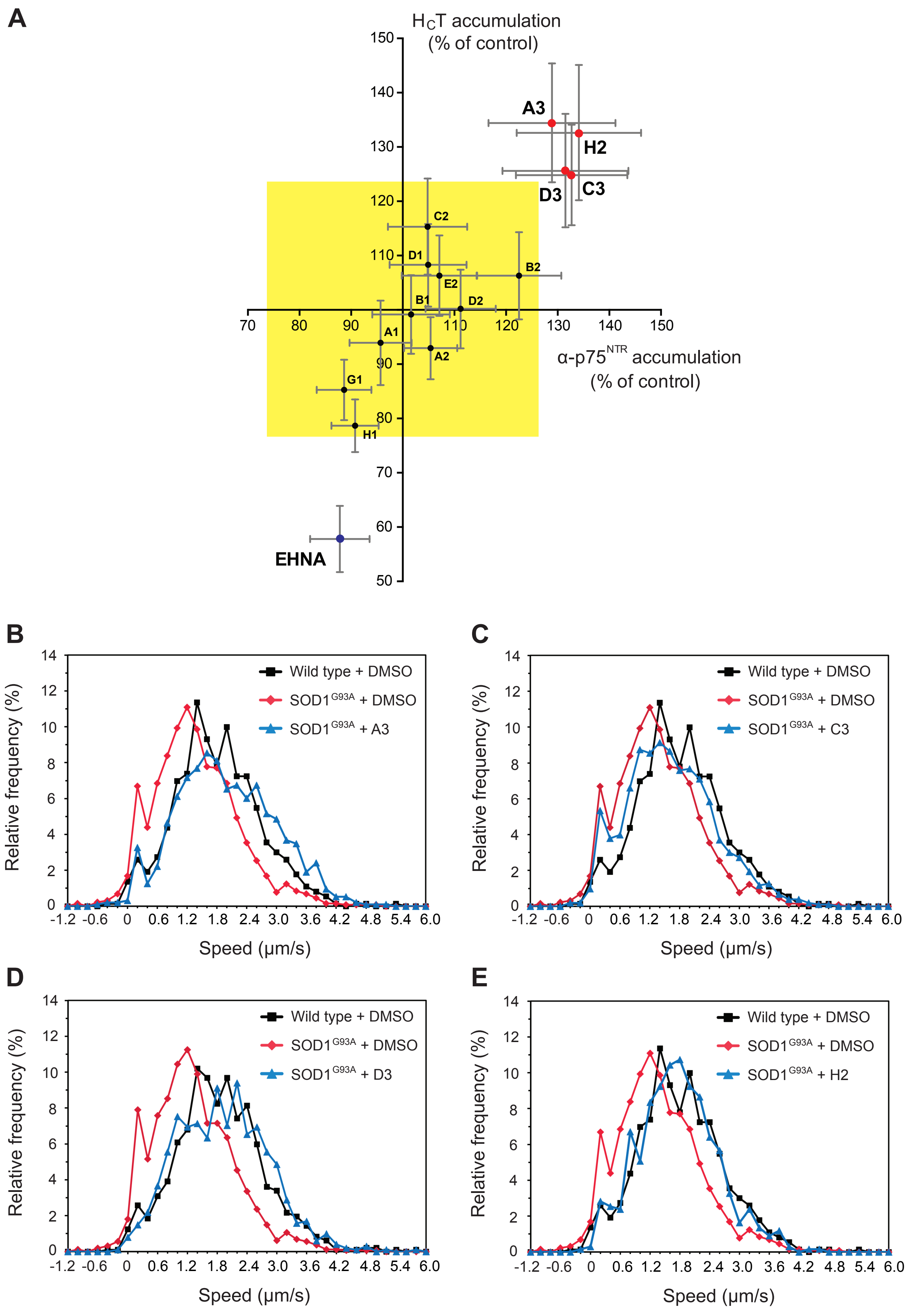
A
HCT accumulation (% of control)
α-p75NTR accumulation (% of control)
A3
H2
D3
C3
C2
D1
E2
B2
B1
D2
A1
A2
G1
H1
EHNA
B
C
D
E
Relative frequency (%)
Speed (μm/s)
Wild type + DMSO
SOD1G93A + DMSO
SOD1G93A + A3
SOD1G93A + C3
SOD1G93A + D3
SOD1G93A + H2

Figure S4

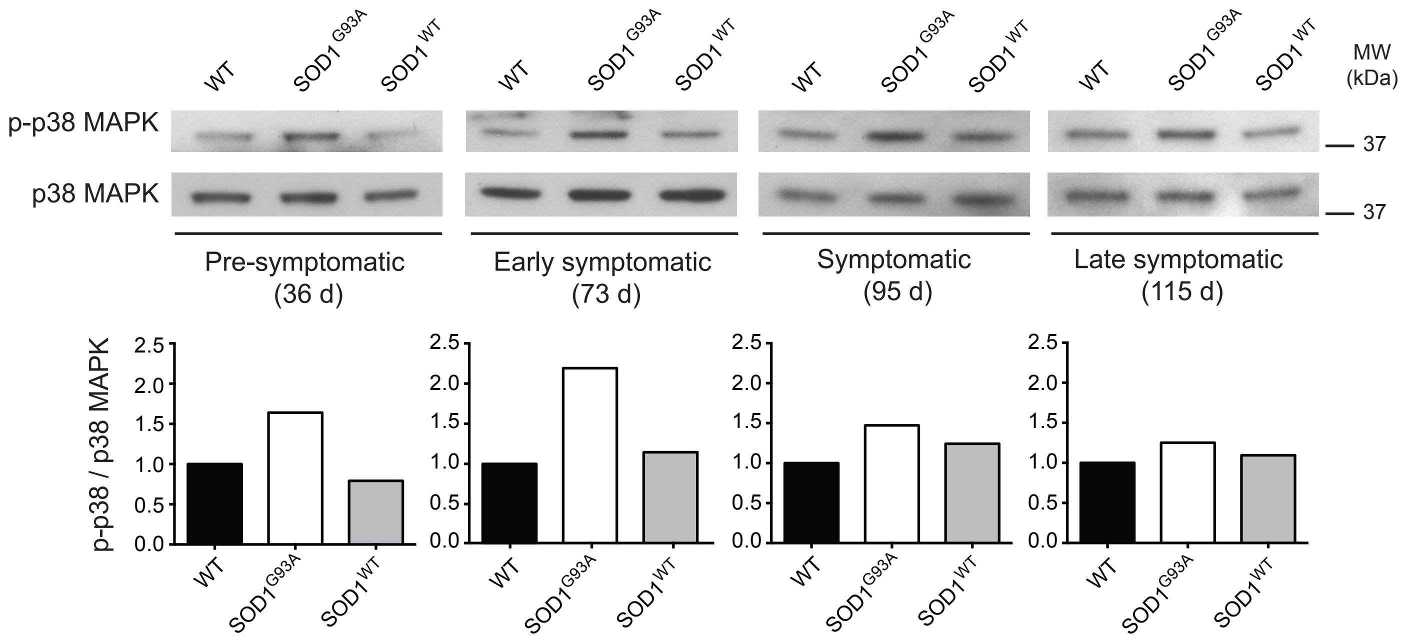

WT
SOD1 G93A
SOD1 WT
MW (kDa)
p-p38 MAPK
p38 MAPK
37
37
Pre-symptomatic (36 d)
Early symptomatic (73 d)
Symptomatic (95 d)
Late symptomatic (115 d)
p-p38 / p38 MAPK
0.0
0.5
1.0
1.5
2.0
2.5
WT
SOD1G93A
SOD1WT

Figure S5

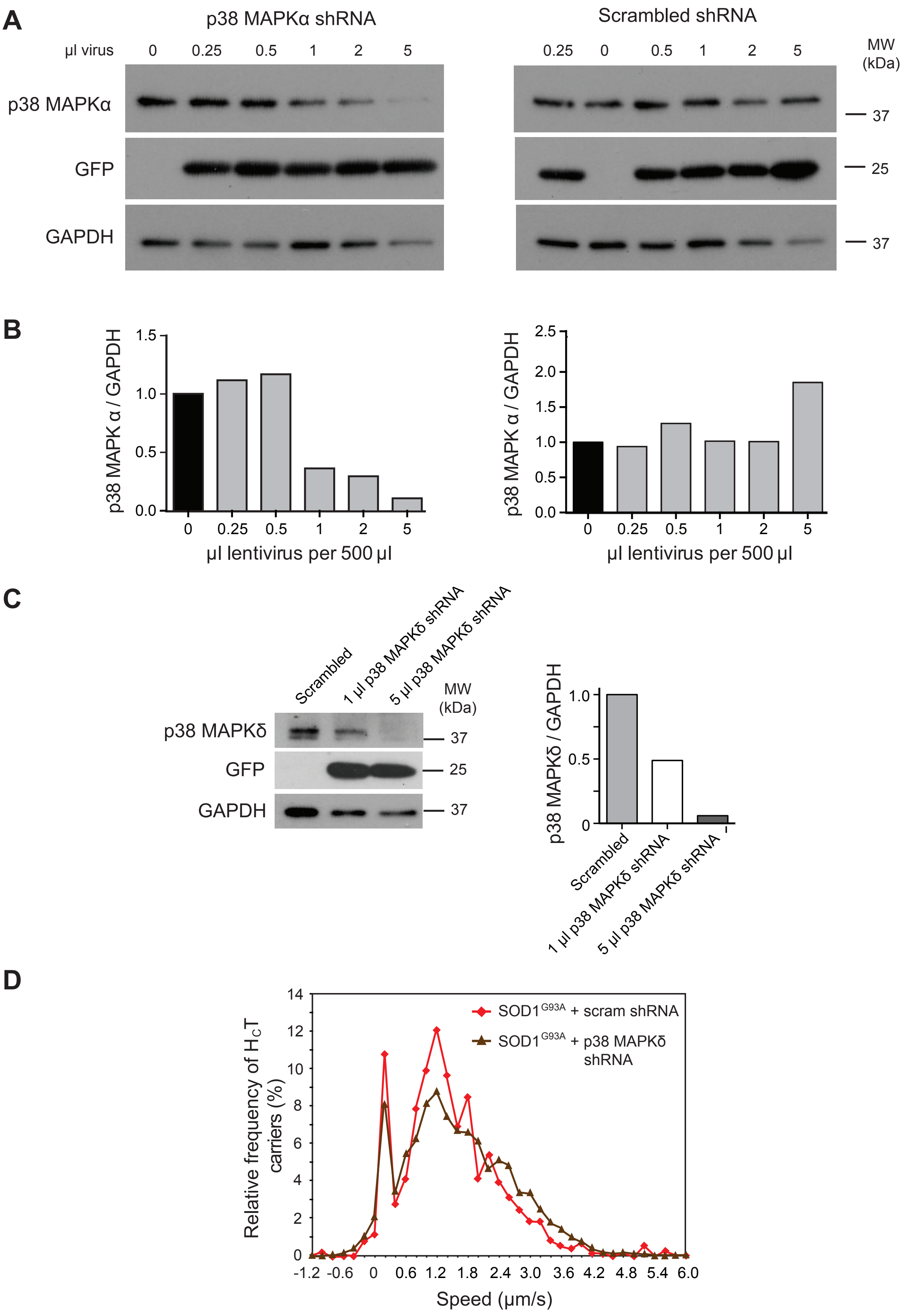

A
p38 MAPKα shRNA
Scrambled shRNA
μl virus
0 0.25 0.5 1 2 5
0.25 0 0.5 1 2 5
MW (kDa)
p38 MAPKα
GFP
GAPDH
37
25
37
B
p38 MAPK α / GAPDH
μl lentivirus per 500 μl
0 0.25 0.5 1 2 5
C
Scrambled
1 μl p38 MAPKδ shRNA
5 μl p38 MAPKδ shRNA
p38 MAPKδ
GFP
GAPDH
MW (kDa)
37
25
37
p38 MAPKδ / GAPDH
D
SOD1^{G93A} + scram shRNA
SOD1^{G93A} + p38 MAPKδ shRNA
Relative frequency of H_CT carriers (%)
Speed (μm/s)

Figure S6

A

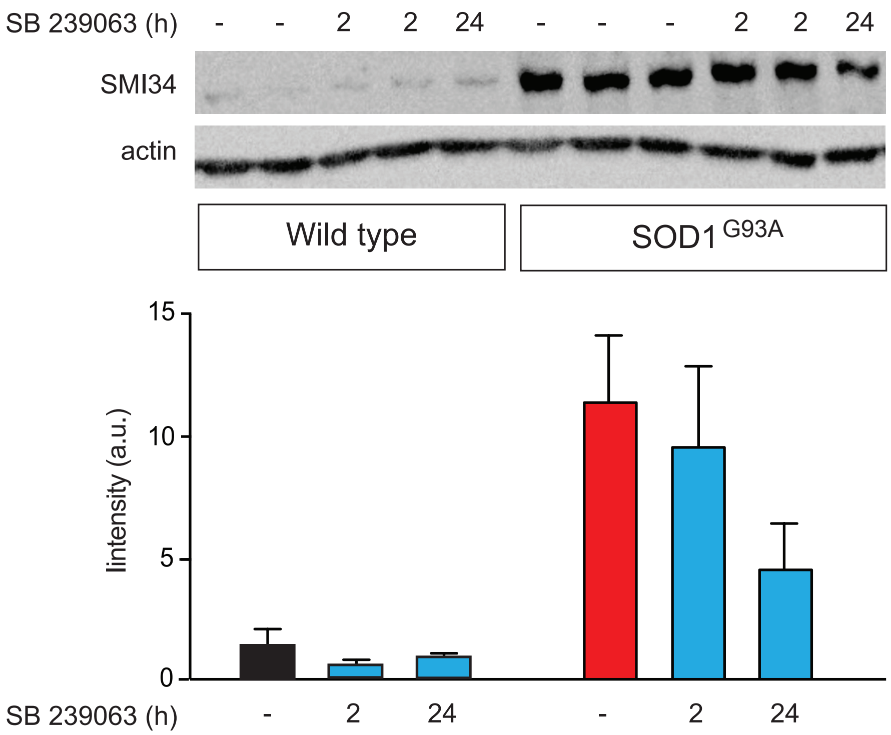

B

Dose (mg/kg)	Time (h)	Brain (μM) ^	Spinal Cord (μM) ^	Muscle (μM) ^^
30	1	0.464	0.405	1.182
30	1	0.555	0.546	1.274
100	1	1.784	1.82	3.241
100	1	1.337	1.379	3.476

C

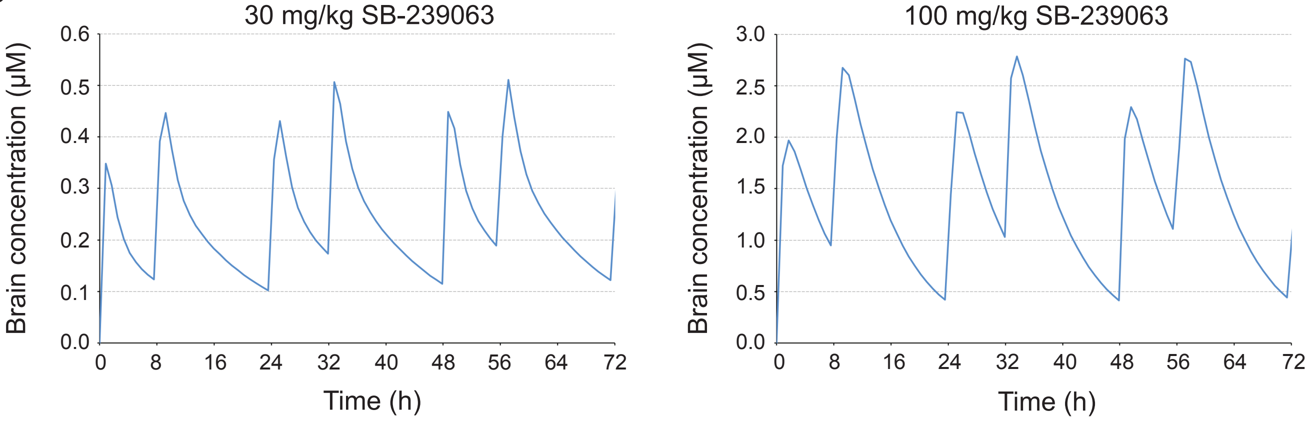

Figure S7

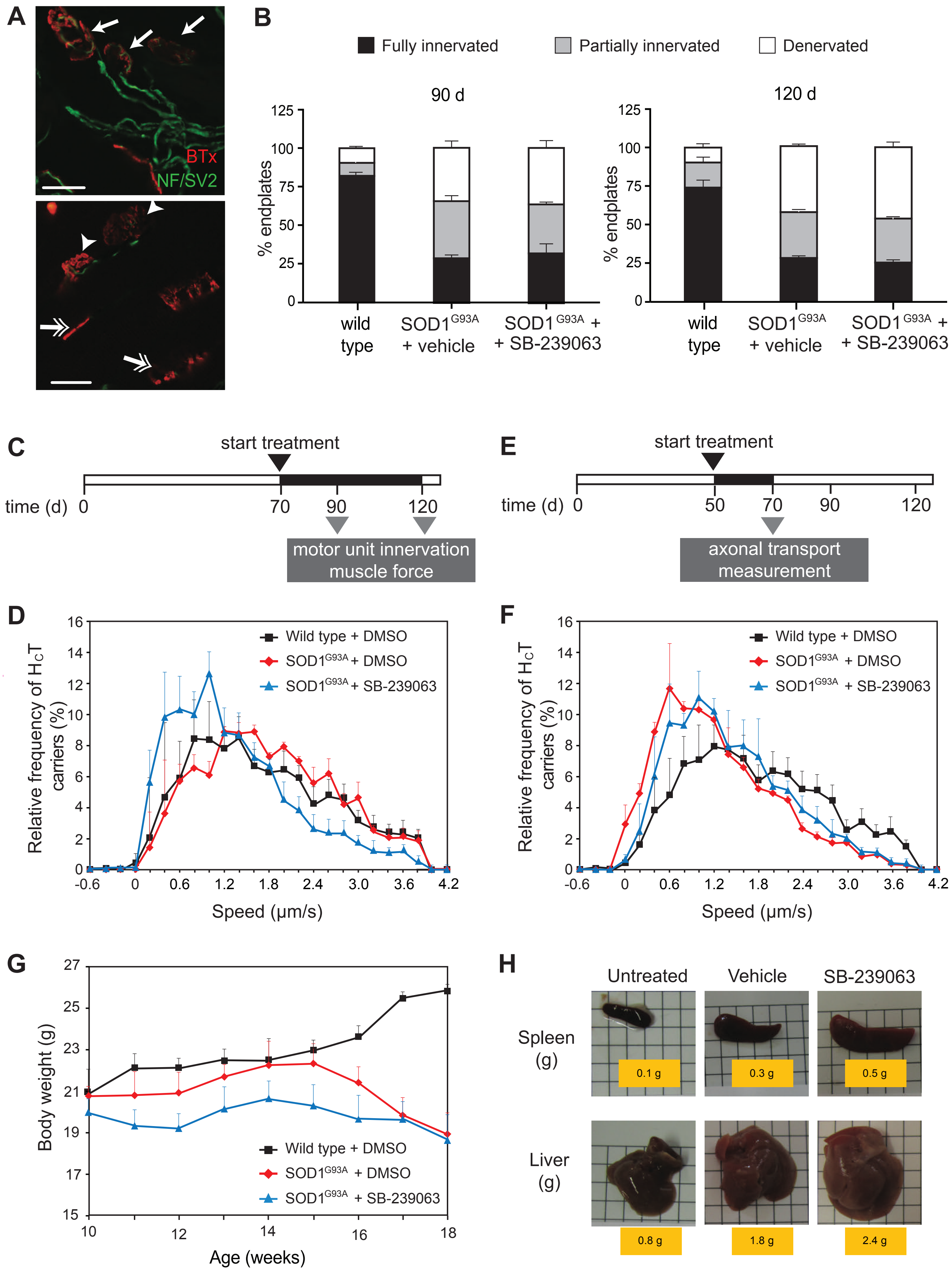

A
BTx
NF/SV2
B
Fully innervated
Partially innervated
Denervated
90 d
120 d
% endplates
wild type
SOD1 G93A + vehicle
SOD1 G93A + SB-239063
C
start treatment
time (d)
0
70
90
120
motor unit innervation
muscle force
E
start treatment
time (d)
0
50
70
90
120
axonal transport
measurement
D
Relative frequency of HcT carriers (%)
Speed (µm/s)
Wild type + DMSO
SOD1 G93A + DMSO
SOD1 G93A + SB-239063
F
Relative frequency of HcT carriers (%)
Speed (µm/s)
Wild type + DMSO
SOD1 G93A + DMSO
SOD1 G93A + SB-239063
G
Body weight (g)
Age (weeks)
Wild type + DMSO
SOD1 G93A + DMSO
SOD1 G93A + SB-239063
H
Untreated
Vehicle
SB-239063
Spleen (g)
0.1 g
0.3 g
0.5 g
Liver (g)
0.8 g
1.8 g
2.4 g
